# Challenges Associated With the Formation of Recombinant Protein Inclusion Bodies in *Escherichia coli* and Strategies to Address Them for Industrial Applications

**DOI:** 10.3389/fbioe.2021.630551

**Published:** 2021-02-10

**Authors:** Arshpreet Bhatwa, Weijun Wang, Yousef I. Hassan, Nadine Abraham, Xiu-Zhen Li, Ting Zhou

**Affiliations:** ^1^Guelph Research and Development Centre, Agriculture and Agri-Food Canada, Guelph, ON, Canada; ^2^Department of Biology, University of Waterloo, Waterloo, ON, Canada; ^3^Department of Molecular and Cellular Biology, University of Guelph, Guelph, ON, Canada

**Keywords:** *E. coli*, protein inclusion bodies, recombinant proteins, protein folding, industrial applications

## Abstract

Recombinant proteins are becoming increasingly important for industrial applications, where *Escherichia coli* is the most widely used bacterial host for their production. However, the formation of inclusion bodies is a frequently encountered challenge for producing soluble and functional recombinant proteins. To overcome this hurdle, different strategies have been developed through adjusting growth conditions, engineering host strains of *E. coli*, altering expression vectors, and modifying the proteins of interest. These approaches will be comprehensively highlighted with some of the new developments in this review. Additionally, the unique features of protein inclusion bodies, the mechanism and influencing factors of their formation, and their potential advantages will also be discussed.

## Introduction

Inclusion bodies (IBs) are nuclear, cytoplasmic, or periplasmic aggregates of bio-macromolecules, mostly proteins. These proteins are generally expressed from foreign or mutated genes without proper post-translational modifications and/or folding (Tsumoto et al., 2003). In humans, protein aggregation has been found associated with numerous protein misfolding diseases (Stirling et al., 2003; Gregersen et al., 2006) such as Huntington (Jimenez-Sanchez et al., 2017), Alzheimer (De Strooper and Karran, 2016), and Parkinson’s diseases (Kalia and Lang, 2015). Besides humans, protein inclusion bodies have been also observed in almost all studied domains of life (e.g., animals, plants, fungi, and bacteria), and they are often related to stress and diseases as well (Blakemore, 1947; Kikkawa and Spitzer, 1969; Espinoza et al., 1991; Li et al., 2009).

Recombinant proteins are becoming increasingly important as enzymes and non-catalytic proteins (e.g., antibodies, hormones, factors, vaccines) for industrial and agricultural applications. *Escherichia coli* is the most popular bacterial host for recombinant proteins production due to: (1) its fast growth rate with a generation time spanning 20 min under optimized conditions (Clark and Maaløe, 1967), (2) well-developed tools of molecular manipulations along with in-depth knowledge of its biology, and (3) the ability to achieve high cell density using inexpensive culture reagents. However, the heterogeneous expression of recombinant proteins in *E. coli* is often hampered by protein aggregation into IBs. This poses a serious challenge for producing soluble recombinant proteins with proper biological function at the laboratory and/or industrial scales. In this review, the mechanism and the factors that influence the formation of recombinant protein IBs will be discussed together with their unique features. In particular, strategies to minimize protein IB formation in *E. coli* will be comprehensively presented in the following sections with the new developments in the field.

## The Mechanisms and the Influencing Factors of Protein Inclusion Bodies Formation in *E. Coli*

**FIGURE 1:**
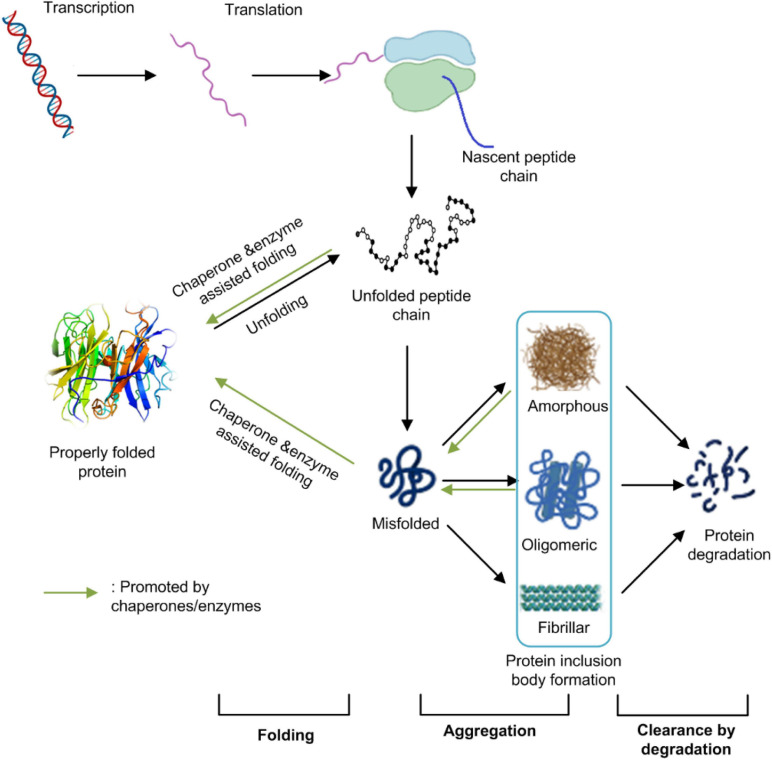
The protein homeostasis network in *E. coli* cells. Protein homeostasis refers to the control of concentration, conformation, binding interactions, and localization of individual proteins making up the proteome by readapting the innate biology of the cell. It involves multiple pieces of cellular machinery for transcription, translation, protein folding, and protein degradation. Protein IBs formation in *E. coli* cells result from an unbalanced equilibrium among protein’s proper folding, aggregation and/or degradation. This figure was generated based on Balch et al. (2008) and Morimoto et al. (2019) with modifications.

All living organisms have evolved a sophisticated mechanism to maintain their protein homeostasis. Protein homeostasis refers to the control of concentration, conformation, binding interactions, and localization of individual proteins making up the proteome by readapting the innate biology of the cell. The maintenance of protein homeostasis is critical for cell function and the overall health of the organism (Balch et al., 2008). It involves multiple pieces of the cellular machinery of transcription, translation, protein post-translational modification, folding, and degradation. Protein IBs formation in *E. coli* cells results from an unbalanced equilibrium among protein proper folding, aggregation, and degradation (Figure 1). It is associated with many factors including host cell metabolism, protein synthesis, and modification machinery, target protein properties, and environmental conditions (Strandberg and Enfors, 1991; Donovan et al., 1996).

IBs formation can be triggered by a high rate of protein expression, which is the pinnacle of recombinant proteins production pipelines. This goal is often achieved by constructing expression vectors with strong promoters (e.g., *T7* and *Lac* promotors), using plasmids with high copy numbers, optimizing codon usage, or engineering *E. coli* host-strains for their fast growth. However, when the rate of a recombinant protein expression exceeds the ability of the host cells to manage protein post-translational modifications and folding, the target protein will be increasingly misfolded, and then aggregated into IBs as the hydrophobic residues buried in the native protein are exposed on its surfaces. Furthermore, host cells cannot sustain protein expression at such high levels due to the cell stress and metabolic burden from the increased energy demand (Gill et al., 2000; Zeng and Yang, 2019).

The lack of proper host machinery for protein post-translational modifications (PTMs) is another factor leading to protein misfolding, particularly when expressing eukaryotic proteins (e.g., mammalian, plant, and fungal proteins) in *E. coli*. PTMs are important to achieve a native, biologically active conformation, and can significantly affect the characteristics of proteins including their charge, hydrophobicity, solvent accessibility, etc. (Walsh, 2010). Among multiple PTMs, glycosylation particularly plays critical roles in protein sorting, folding, localization, and quality control in eukaryotic organisms (Molinari, 2007). Yet another PTM, disulfide bond formation, is also crucial for producing stable proteins. *E. coli* lacks subcellular compartments such as the endoplasmic reticulum and Golgi apparatus to facilitate glycosylation and disulfide bridge formation. Secreting recombinant protein to the oxidative environment of *E. coli* periplasm has proven successful for disulfide bond formation in certain cases (Baumgarten et al., 2018; Zhang et al., 2018). However, secretion efficiency is often limited by the size and structure of the protein of interest as well as the secretory machinery of *E. coli* (Choi et al., 2000; Choi and Lee, 2004).

Protein aggregation into inclusion bodies is also governed by physicochemical and structural features of the proteins themselves. These characteristics include the molecular weight, the number of contiguous hydrophobic residues, and low complexity regions (Dyson et al., 2004; Goh et al., 2004). Dyson et al. (2004) used a deep-mining approach to investigate the correlation between these factors and the possibility to produce soluble functional mammalian proteins in *E. coli*. Generally, single-domain proteins with low molecular weight tend to be produced in soluble form with retention of proteins functionality. This is partly due to the requirement for fewer folding intermediates in the protein folding pathway (Markossian and Kurganov, 2004). Contrary to this, the folding of multi-domain proteins may be accompanied by folded or misfolded intermediates increasing the likelihood of aggregation. Other aggregation-prone proteins such as intrinsically disordered proteins and membrane proteins also pose significant challenges in protein expression studies. The presence of disordered, low complexity regions and stretches of hydrophobic regions predispose these proteins to IBs formation.

Environmental conditions, such as culture temperature and pH, also affect IBs formation during recombinant proteins production in *E. coli.* The effect of heat stress at 45°C on inducing the aggregation of recombinant luciferases in *E. coli* has been reported earlier (Winkler et al., 2010), as well as the ability of physiological pH conditions (pH = 7.5) to leverage a beneficial effect on the heterologous expression of *Boophilus microplus* sphingomyelinase-D in *E. coli* (Castellanos-Mendoza et al., 2014). Due the above reasoning, tailoring culture conditions is an approach that is often used as a solution to minimize IBs formation of recombinant proteins in *E. coli.*

In essence, the aggregation process is driven by hydrophobic interactions that act as countermeasures shielding hydrophobic stretches of protein from the surrounding aqueous environment (Winkler et al., 2010). Newly formed aggregates may then promote nucleation by acting as seeds for the aggregation of other highly similar proteins (Morell et al., 2008). This process is more likely to occur in prokaryotes such as *E. coli*, which do not possess the appropriate protein-modification machinery. High levels of expression coupled with a lack of modification machinery promote misfolding, resulting in the inadvertent exposure of hydrophobic residues on the protein surface (Markossian and Kurganov, 2004; Schramm et al., 2019).

## The Unique Features of Protein Inclusion Bodies in *E. Coli*

Relative to soluble proteins, IBs proteins in *E. coli* display unique characteristics in terms of structure, morphology, and composition. Morphologically, these aggregates are observed as dense refractile particles with smooth or irregular rough surfaces (Carriö et al., 2000). These characteristics may vary from protein to protein and are dependent on the properties of the expressed proteins (Carriö et al., 2000).

Protein aggregates may consist of ordered structures termed amyloid fibrils or disordered, amorphous structures as observed for regular IBs proteins (Bowden et al., 1991; Wang, 2009). Amorphous aggregation is a thermodynamically favorable process obviating the need for a high energy barrier necessary for nucleation processes such as amyloid fibrillation and crystallization (Yoshimura et al., 2012). The catalytic activity were found for some amyloid aggregates which are characterized by cross β-sheet motifs (Chapman et al., 2002; Carrió et al., 2005; Jordal et al., 2009; Upadhyay et al., 2012; Singh et al., 2020). Conversely, *E. coli* protein IBs appear as amorphous aggregates lacking proper structure and function. Despite this, a growing body of evidence has suggested that certain IBs proteins may possess amyloid-like structures with an associated functionality (Carrió et al., 2005; Upadhyay et al., 2012; Singh et al., 2020). Amyloid-like properties were seen in β-galactosidase IBs expressed in *E. coli*, which were shown to be biologically active (Carrió et al., 2005). Another study carried out by Singh et al. (2020) to characterize asparaginase IBs confirmed the high β-content as well as the catalytic activity of the amyloid-like aggregates. Furthermore, it was determined that temperature could modulate the levels of reported amyloid features (Singh et al., 2020). The parallel findings were reported in a similar study (Žerovnik et al., 2007). The mechanism behind how temperature and time may influence IBs formation/structure is not well understood and speculations of protein-specific outcomes do exist (Upadhyay et al., 2012).

*E. coli* IBs while largely composed of self-aggregated protein, also contain traces of other bio-macromolecules including nucleic acids (Krachmarova et al., 2020), and/or phospholipids (Valax and Georgiou, 1993). Within the biotechnological context, proteins in IBs predominantly consist of expressed foreign proteins (Rinas and Bailey, 1992; Valax and Georgiou, 1993). The nature of such highly specific protein self-aggregates was revealed through a pioneering study using two aggregation-prone proteins that were fluorescently labeled, namely the ΔF508 mutant of the cystic fibrosis transmembrane conductance regulator (F508) and the P23H rhodopsin mutant (P23H). Interestingly, the aggregation of P23H with other aggregation-prone proteins was not observed and P23H was seen aggregating predominantly with itself (Rajan et al., 2001). This phenomenon was further validated by an experiment conducted thorough the co-expression of Aβ42 amyloid peptide with VP1 capsid protein from the foot-and-mouth disease virus. The Aβ42 amyloid peptide only co-aggregated with itself and was not observed aggregating with VP1 (Morell et al., 2008). Given the highly homogenous protein composition, *E. coli* protein IBs provide a unique source of an almost pure target protein and might be further exploited for biotechnological applications (see “Potential Advantages of Protein Inclusion Bodies in Industrial Applications” section for further discussion).

Bacterial IBs also show specific cellular distribution at the poles and/or septation sites of *E. coli* cells. The majority of IBs are formed near the poles, and 24% of formed IBs showed a migratory movement after formation (Winkler et al., 2010). Similarly, a VP1 capsid protein tagged with green fluorescent protein aggregated specifically at *E. coli* cell poles (Rueda et al., 2014). The results of the above studies suggest that IBs are exclusively assembled at the cellular pole(s) or are transported thereafter the formation event. Conceivably, this polar distribution of IBs will result in an asymmetrical pattern of inheritance during cell division with one daughter cell containing IBs while the other is free. This distribution pattern is beneficial for the later daughter cells as it places less burden with reduced or no inclusion bodies (Lindner et al., 2008). Notably, daughter cells that inherited the new poles without inclusion bodies showed higher growth rates compared to the cells carrying the poles with IBs (Winkler et al., 2010).

A direct consequence of light scattering by protein IBs is that the *E. coli* lysate often appears as a milk-like broth when the recombinant protein forms IBs are released. This was often used as a primary indicator of IBs formation in the many protein expression trials of several research groups. Under light microscopy, protein IBs often appear as dense refractile particles with varying sizes inside cells (Martelli and Castellano, 1971; Katoh et al., 2006). Additionally, SDS-PAGE analysis of soluble and insoluble fractions of bacterial cell lysates can also be applied to test the presence of IBs of the expressed protein(s). This can be visualized by a strongly stained protein band corresponding to the molecular size of the target protein in the insoluble fraction of the cell lysate. Amyloid-protein aggregates can also be identified by using dyes such as Congo Red (Georgalis et al., 1998; Yakupova et al., 2019). Congo red shows apple-green birefringence under the light microscope. Molecular motor dyes such as thioflavin-T (Biancalana and Koide, 2010) and Proteostat (Navarro and Ventura, 2014) may also be used for visualization. These types of dyes show fluorescence enhancement when rotary movement is constricted in microenvironments (Shen et al., 2011; Navarro and Ventura, 2014).

## Strategies to Minimize Protein Inclusion Bodies Formation

Based on the above-discussed mechanisms governing proteins’ IBs formation, many strategies have been developed recently to minimize this phenomenon. In general, these strategies contribute to one or more of the following aspects (Table 1): (1) reducing protein synthesis rate, (2) inducing the production of endogenous chaperones, and/or the synthesis or the absorption of osmolytes in *E. coli* cells, (3) introducing additional cellular components (e.g., chaperones and foldases) to *E. coli* cells or adding chemical chaperones to the culture medium to assist protein folding and modification, and (4) modifying the protein of interest by removing structural elements contributing to protein IBs formation, and/or fusing the target protein to a soluble protein or peptide tags. The effectiveness of each approach may vary from one protein to another and it requires an empirical optimization. These strategies will be elaborated below with solid examples (Table 1).

**TABLE 1:** The various strategies to control and minimize the formation of recombinant protein inclusion bodies in *E. coli*.

Strategies	Specific approaches	Potential mechanism	Comments	References
Tailoring culture conditions	Lowering the culture temperature in induction phase	Reducing protein expression rate	Two-phase culture used. First-phase at 37°C for cell growth, second phase at 15–20°C for the induction of protein expression	Cabilly, 1989; Shirano and Shibata, 1990; Jung et al., 2013; Sina et al., 2015; Carere et al., 2018a; Wang et al., 2019
	Introducing a short time heat shock prior to expression induction	To induce chaperons’ production, meanwhile minimize IBs formation	E.g., 47°C for 20–30 min	Oganesyan et al., 2007
	Decreasing the concentration of inducer (e.g., IPTG)	Reducing protein expression rate	E.g., 0.01–0.05 mM instead of 0.5–1.0 mM	Jhamb and Sahoo, 2012; Sina et al., 2015
	Adding glucose in growth medium	Reducing protein expression rate through catabolic repression effect of glucose to the induction	The glucose concentration at 1–2% was often used	Grossman et al., 1998
	Adding chemical additives (e.g., D-sorbitol, glycerol, ethanol, NaCl et al)	Sorbitol, glycerol and NaCl will cause osmotic stress and further induce osmolytes synthesis or uptake. Ethanol will elicit heat shock response and induce the production of chaperones	Often used conditions: Sorbitol (0.5–1.0 M), NaCl (0.2–0.8 M), Betaine (1 mM), Ethanol [3% (v/v)]	Blackwell and Horgan, 1991; Diamant et al., 2001; Oganesyan et al., 2007
	Adding co-factors of target protein in growth medium	To assist proper protein folding	Many proteins require cofactors for their proper folding such as metalloenzymes	Bushmarina et al., 2006; Rosano and Ceccarelli, 2014
	Use buffer to control pH of growth medium	Controlling the pH fluctuation for the proper protonation states of proteins	No fluctuations to the protein, keeps it chemically stable	Castellanos-Mendoza et al., 2014
Expression host engineering	Engineered strains to catalyze di-sulfide bond formation—*TrxB*, *gor* mutants, CyDisCo system	*Trxb*^–^ and *gor*^–^ generate a more oxidizing environment. CyDisCo involves di-sulfide bonds catalyzed by a sulfhydryl oxidase Erv1p	Proteins requiring di-sulfide bonds can be successfully folded and functional. E.g., SHuffle and Origami strains, CyDisCo system	Xiong et al., 2005; Rasiah and Rehm, 2009; Nguyen et al., 2011; Lobstein et al., 2012; Hatahet and Ruddock, 2013
	Engineering strains to perform glycosylation	Addition of enzymes or pathways able to catalyze N- or O-linked glycosylation Knockouts of *wecA* and *waaL* to remove competing glycan pathways	Important implications for activity, structure, and stability E.g., CLM37 and CLM24 strains	Wacker et al., 2002; Feldman et al., 2005
	Co-expressing chaperone	Aid in the proper protein folding	E.g., GroEL, GroES, ClpB	Lee et al., 2004; de Marco et al., 2007; Jhamb and Sahoo, 2012
	Co-expressing foldase	Aid in the proper protein folding and disulfide bond formation	Include protein disulfide isomerases (PDI) and peptidyl prolyl isomerases (PPI)	Ngiam et al., 2000; Lee et al., 2004; Jung et al., 2013; Zhuo et al., 2014
	Strains engineered for membrane proteins or toxic proteins	Dampening of T7 RNA polymerase expression and/or activity	Aims to reduce expression levels to reduce toxicity and improve membrane protein expression E.g., *E. coli* strains C41 (DE3), C43 (DE3), Lemo21 (DE3), BL21 (DE3) pLysS, pAVEway^TM^	Miroux and Walker, 1996; Wagner et al., 2008; Kwon et al., 2015; Kim et al., 2017
	Co-expressing multiple components of protein complex	The co-expression of protein components is beneficial for protein folding, stability and protect individual components from degradation	Using compatible duet vectors with different antibiotics resistance	Tolia and Joshua-Tor, 2006
	Engineered metal ion transport for metalloenzymes	Overexpress operons involved in uptake/transport of metal cofactors	Overexpressing cobalamin transport pathways and Suf pathways shown to produce proteins with full iron occupancy	Lanz et al., 2018; Corless et al., 2020
	Use weaker promotor	Reducing protein expression rate	Better balance between protein synthesis and folding, and lower metabolic burden to host cells	Kaur et al., 2018
	Linked to a soluble fusion tag or chaperone at either N- or C-terminus.	Improve protein expression yields, solubility and folding, facilitate protein purification.	E.g., maltose binding protein, glutathione-S-transferase, Spy	Vu et al., 2014; Ruan et al., 2020
Altering expression vector	Plasmid display technology, linking the target protein to a DBD	Target protein and DBD are attached to the plasmid itself, aids in stabilization	Ensure a soluble DBD partner E.g., Oct-1 DBD, GAL4 DBD	Xiong et al., 2005; Park et al., 2013, 2020
	Use a low copy number plasmid	Reducing protein expression rate	Better balance between protein synthesis and folding, and lower metabolic burden to host	Kaur et al., 2018
	Minimize the hydrophobic patch on the surface of protein	Site directed mutagenesis to change aggregation-promoting residues	Prediction using programs Ex. TANGO, PASTA 2.0, AMYLPRED 2.0, Protein-Sol, SoDoPE	Conchillo-Solé et al., 2007; Tsolis et al., 2013; Walsh et al., 2014; Hebditch et al., 2017; Bhandari et al., 2020
	Express partial protein (truncated and soluble domain)	Potential aggregation prone protein is expressed in a soluble state	Based on the purpose for the protein, as it may not be functional	Chen et al., 2003
Modifying the protein of interest	Add signal peptide to direct the expressed protein into periplasmic area	It is beneficial for folding with the more oxidized environment and foldases in the periplasmic space	Less proteolytic activity in periplasmic space	Dow et al., 2015; Malik, 2016

### Tailoring Bacterial Culture Conditions

A reduction in the level of IBs formation can be achieved by modifying culture conditions, including growth temperature, inducer concentrations, and culture additives. These factors often influence the rate of protein expression, and aid in the protein folding process in *E. coli* cells (Table 1).

Lowering culture temperatures below the optimal 37°C can decrease the rate of *E. coli* cells growth and enhance their protein expression in a soluble form (Cabilly, 1989; Shirano and Shibata, 1990; Yang and Zhang, 2013). This approach is usually carried out in two phases, where the first-phase culture at 37°C is optimal for the growth of *E. coli* to reach a high cell density. Second-phase temperatures are set to low ones (e.g., 15–20°C) for the protein expression induction with a low rate. Such an approach has been very effective in our efforts to produce multiple soluble and active enzymes of agricultural importance, including Tri101 acetyltransferase (Hassan et al., 2016), DepA (Carere et al., 2018a), and DepB (Carere et al., 2018b) for mycotoxins detoxification in addition to several carbohydrate-active enzymes (CAZymes) for agricultural biomass valorization (Wang et al., 2018, 2019). In contrast, culture temperatures greater than 37°C have been also scrutinized in the past for their effect on protein expression, as the heat shock could presumptively induce the production of molecular chaperones (Hoffmann and Rinas, 2000; Kim et al., 2013). However, the use of high cultivation temperatures (>37°C) generally resulted in elevated inclusion bodies formation, likely due to the increase in hydrophobic interactions (Restrepo-Pineda et al., 2019), as well as cellular stress poised by protein denaturation at temperatures greater than 37°C (de Groot and Ventura, 2006). To take the advantage of the production of the molecular chaperones, induced by high temperatures, while minimizing the formation of IBs, a short heat shock (47°C, 30 min) was used to treat *E. coli* cells before inducing protein expression under 20°C. This culture program improved the expression of several recombinant proteins into their soluble form (Oganesyan et al., 2007).

Protein expression rates can also be reduced by lowering inducer concentrations or adding glucose into the culture medium (Table 1). In *E. coli* protein expression systems, the *lac* operon has been extensively tailored for the induction of heterogenous proteins expression, where lactose is an activator of the *lac* operon (Browning et al., 2019). Isopropyl β-D-1-thiogalactopyranoside (IPTG) is a structural analog of allolactose, a lactose metabolite, and therefore efficiently activates the *lac* operon through binding to lac repressor protein. However, since IPTG is not easily metabolized, its concentration remains consistent compared to lactose in bacterial cultures. Therefore, IPTG has been a widely used chemical inducer for recombinant proteins expression (Donovan et al., 1996; Lewis, 2013). Reducing IPTG concentrations in the culture medium can decrease protein expression rates to manageable levels without placing a metabolic burden on *E. coli* cells, which is ultimately favorable for the proper folding of recombinant proteins (Donovan et al., 1996). Alternatively, adding glucose to the medium can also decrease the rate of protein expression through competitive catabolic repression. Glucose inhibits adenylate cyclase (AC) activity on synthesizing cAMP from ATP. The cAMP is able to regulate the *lac* operon by binding to the catabolic activator protein (CAP). The latter then binds to the promoter CAP site and further stimulates the binding of RNA polymerase to the promoter region to initiate transcription. In the presence of glucose, low levels of cAMP lead to a low level of CAP activation, resulting in a low transcription level for the target gene (Lewis, 2013).

Some chemical additives in culture media display beneficial effects on the expression of soluble recombinant proteins. These additives generally include osmotic stress-triggering chemicals, heat shock-inducing chemicals, and protein cofactors. For example, the presence of salt or sorbitol in the culture medium facilitated the expression of target proteins in their soluble form by triggering osmotic stress and stimulating osmolyte (e.g., betaine, trehalose) synthesis/uptake in *E. coli* cells (Blackwell and Horgan, 1991; Diamant et al., 2001; Oganesyan et al., 2007). These osmolytes can act as “chemical chaperones” by increasing the stability of native proteins and assisting in the refolding of unfolded polypeptides (Diamant et al., 2001; Papp and Csermely, 2006). This group of osmotic stress-triggering chemicals could also include glycerol, mannitol, and other polyols. Within them, D-sorbitol (along with betaine) is the most widely used additive in minimizing recombinant proteins IBs formation. D-sorbitol displayed beneficial effects in our earlier efforts to produce several CAZymes for their functional and structural characterization, and their application in agricultural by-products valorization (Wang et al., 2016, 2018; Sarch et al., 2019). The inclusion of ethanol in culture media (e.g., 3%, v/v) has been reported to enhance the solubilities of some recombinant proteins (Kusano et al., 1999). Ethanol here elicits a heat shock response and induces the production of chaperones. Additionally, adding co-factors to the growth media could be another consideration to improve protein expression in soluble form, as many proteins require cofactors for their proper folding and function (Bushmarina et al., 2006).

The pH of bacterial cultures often affects the charges of present proteins and can further impact their properties. For instance, pH has been reported to affect the tendency of β peptides to form amyloid-like structures *in vitro* (Castellanos-Mendoza et al., 2014), suggesting that pH might be an important factor that can significantly influence the levels of IBs formation. Mendoza-Castellanos’s group compared the effects of controlled and uncontrolled pH conditions on the formation of sphingomyelinase-D IBs when grown in a super broth medium (Castellanos-Mendoza et al., 2014). The results demonstrated that protein’s aggregation occurred at higher levels under uncontrolled pH condition (as pH fluctuated with cellular metabolism) than controlled ones, where the controlled pH environment (pH = 7.5) was achieved by coupling with an automatic supplementation of 1.0 M NaOH (when needed). Therefore, keeping culture pH stable at a certain level could be beneficial in minimizing the formation of recombinant protein IBs in *E. coli*.

Taken together, it is worth noting that altering culture conditions usually present the simplest solution to reduce IBs formation in *E. coli*. However, culture conditions favorable for soluble protein production may vary depending on the involved proteins of interest and the used host strains of *E. coli*, and thus require experimental optimization.

### Host Engineering for Recombinant Protein Expression in Soluble Form

Recent advancements in biotechnology and engineering have led to the development of many efficient and flexible systems for recombinant proteins expression/production in *E. coli*. In this section, we address a few engineered *E. coli* strains to aid disulfide bond formation, glycoengineered strains for glycosylation, strains which offer fine tuning over protein expression, expression of chaperones and finally we briefly mention host engineering for certain metallo-enzymes (Table 1).

#### To Improve Disulfide Bond Formation

**FIGURE 2:**
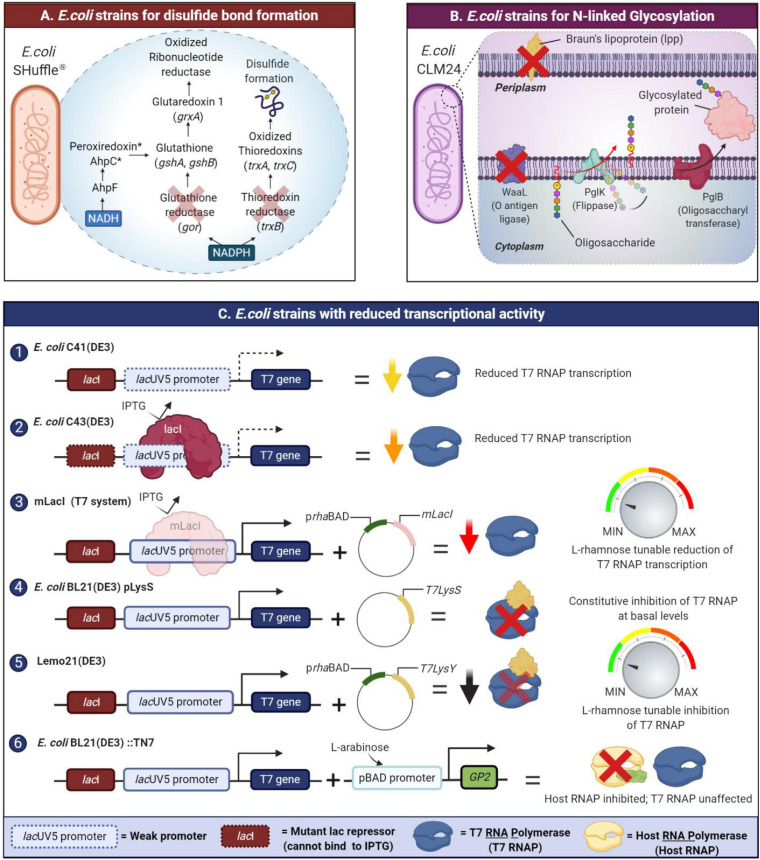
New developments in *E. coli* strain’s engineering to tackle the issue of IBs formation in recombinant protein expression. Amongst the issues is its inability to catalyze di-sulfide bond formation, glycosylation, and the high strength of expression. **(A)** SHuffle strains enable disulfide bond formation by oxidized thioredoxins and mutant AhpC* transfers electrons to GSH/glutaredoxin pathway allowing for the reduction of oxidized ribonucleotide reductase which is essential for growth. **(B)** N- or O-glycosylation in glycoengineered *E. coli* is conducted by the protein glycosylation (*pgl*) locus which is responsible for the biosynthesis of the glycans. Glycoengineered strains like *E. coli* CLM24 could potentially be engineered to be “leaky” facilitating secretion of the glycoprotein to culture media. As proof of concept, *E. coli* CLM37 was engineered to be “leaky” by deleting the Braun’s lipoprotein (*lpp* gene) which connects the outer membrane to the peptidoglycan layer. **(C)** Various *E. coli* strains have been engineered (1, 2, 3) to modulate transcription or (4, 5) inhibition of either orthogonal T7 RNA polymerases (RNAP). There are also those strains engineered in (6) decoupling of host cell growth from recombinant protein production via inhibition of *E. coli* RNAPs.

Many recombinant proteins require correct disulfide bonding to attain their biological functionality. In *E. coli*, the disulfide bond formation often occurs in the periplasm through the oxidation of a pair of cysteine residues. In contrast, the S-S bond formation in the cytoplasm is disfavored due to the existence of a reducing environment maintained by two separate thioredoxin reductase (trxB) and glutaredoxin reductase (gor) systems (Francis and Page, 2010). To attain the correct disulfide bonding of recombinant proteins expressed in the cytoplasm of *E. coli*, specific strains with a unique trio of modifications have been engineered to a more oxidative environment by eliminating glutathione reductase (*gor* gene) and thioredoxin reductase (*trxB* gene) along with a suppressor mutation in the *ahpC* gene (Bessette et al., 1999). These strains include commercially available Origami and SHuffle strains (Rasiah and Rehm, 2009; Lobstein et al., 2012). The SHuffle strain has both mutations of the Origami strain but also constitutively expresses a disulfide bond isomerase which promotes the correction of mis-oxidized proteins (Lobstein et al., 2012; Figure 2A). Another alternative to these strains is the CyDisCo system, which enables the expression of disulfide bond containing proteins in the cytoplasm of *E. coli* without modifications to endogenous reducing pathways. Here, *de novo* disulfide bond formation is catalyzed by Erv1p, a eukaryotic sulfhydryl oxidase (Nguyen et al., 2011; Hatahet and Ruddock, 2013) while a protein disulfide isomerase (PDI) rectifies errors in disulfide bond formation (Gaciarz et al., 2017). Although a CyDisCo system could be constructed using periplasmic *E. coli* DsbB or vitamin K epoxide reductase, these transmembrane proteins require extensive engineering to invert their membrane topology to function stably in the cytoplasm (Hatahet and Ruddock, 2013). Recently, CyDisCo was benchmarked by expressing domain constructs of mammalian extracellular matrix proteins including mucin-2 (MUC2), alpha tectorin (TECTA) which possess von Willebrand Factor D domains implicated in several human diseases (Sohail et al., 2020). Both proteins were successfully purified in a soluble state; however, the only limitation to this system appears to be the number of disulfide rich regions in the expressed protein.

#### Optimized Glycoengineered *E. coli* Strains for N-Linked Glycosylation

Protein glycosylation is a post-translational modification that involves the addition of glycans to proteins. Glycosylation has important implications for the folding, activity, structure, solubility, and stability of the protein (Gomes et al., 2016). Two forms of glycosylation exist: O-linked glycosylation, found in bacteria and N-linked glycosylation which is a rare occurrence for bacteria and more exclusive to the eukaryotic and archaea domain (Wacker et al., 2002; Chen, 2012). Prokaryotes like *E. coli* have difficulty in processing N-linked glycosylation, and mistakes often lead to the formation of aggregation-prone proteins. To accommodate the lack of N-glycosylation machinery, engineering *E. coli* strains with specific enzymes able to catalyze glycosylation presents a promising solution (Figure 2B). PglB, an oligosaccharyl transferase, is a key enzyme for the N-glycosylation pathway in *Campylobacter jejuni* where its function is to transfer an O antigen or O-polysaccharide to an L-asparagine residue on the acceptor protein. As proof of concept, PglB was co-expressed in a host plasmid, enabling the transfer of an O polysaccharide from a lipid carrier to the model protein, AcrA (Wacker et al., 2002; Feldman et al., 2005). This alternative allows for proper glycosylation to take place as well as ensuring that expressed recombinant proteins properly fold. Optimized *E. coli* strains for glycoprotein production include *E. coli* CLM37 and *E. coli* CLM24, which possess knockouts of *wecA* and *waaL* genes to remove competing glycan pathways present and reduce the metabolic load due to expression of plasmids containing the PglB gene. Several research groups have also employed “leaky” glycoengineered *E. coli* strains. In this context, the term “leaky” refers to strains with increased permeability of the outer membrane. These strains possess mutations or knockouts of the *lpp* gene which encodes a key lipoprotein (lpp), often referred to as Braun’s lipoprotein. In *E. coli*, lpp maintains connectivity between the outer membrane and the peptidoglycan layer through covalent interactions. Prior studies revealed that *E. coli lpp* mutants (*E. coli* E609YΔ*lpp*, *E. coli* JM109 (DE3)Δ*lpp*) have increased permeability of the outer membrane which is advantageous for recombinant production of glycoproteins including therapeutic proteins including glycosylated antibody fragments. One caveat that limits the use of such “leaky” host systems is the size of the protein as well as the dynamics of the secretion system in question (Ni et al., 2007; Shin and Chen, 2008). Ding et al. (2019) introduced the *lpp* mutation into a glycoprotein engineered *E. coli* strain of CLM37 to produce N-glycosylated anti-VEGFR2 (vascular endothelial growth factor receptor 2). Levels of the secreted protein were at least 11–15 times higher compared to the parent *E. coli* CLM37 strain (no *lpp* deletion) with yields of 70 ± 3.4 mg/L (Ding et al., 2019).

Another proprietary “leaky” strain, *E. coli* enGenes-X-press^TM^ possesses an expression system which incorporates the expression system described by Stargardt et al. (2020) and enables delivery of the recombinant protein to the culture medium which streamlines downstream processing steps.

#### Accessory Proteins to Correct Misfolding: Foldases and Chaperones

Foldases are a group of proteins whose function is to assist in the proper folding of proteins (Kim et al., 2013). Important foldases include thiol-disulfide oxidoreductase and protein disulfide isomerases (PDI) responsible for disulfide bonds formation, and peptidyl-prolyl isomerases (PPI) catalyzing the cis-trans isomerization of peptide bonds N−terminal to proline (Pro) residues within polypeptide chains. The purpose of co-expressing PDIs and PPIs in *E. coli* is to accelerate the rate-limiting step that leads to errors in protein folding. PDIs are found in eukaryotes as well as the periplasmic space of bacteria. Examples of bacterial PDIs include DsbA, DsbC, and DsbG. For instance, the co-expression of DsbA, DsbC, was shown to greatly enhance target protein solubility (Ngiam et al., 2000; Zhuo et al., 2014). PDIs (e.g., DsbG) also display chaperone-like activities in aiding protein folding (Bothmann and Plückthun, 2000; Shao et al., 2000). Moreover, PPIs were reported to help directly in addressing the above issue by catalyzing isomerization (Stull et al., 2018).

Chaperones are a group of proteins whose function is to stabilize unfolded proteins, unfold them for translocation across membranes or degradation, and/or to assist in their proper folding and assembly (Kim et al., 2013). In *E. coli*, the co-expression of chaperone systems has been shown to improve protein solubility and enhance proteins correct folding, hence leading to reduced IBs accumulation (Thomas and Baneyx, 1996; Lee et al., 2004). Specifically, plasmids harboring genes of the following chaperones: GroEL/GroES, DnaK/DnaJ/GrepE (KJE), ClpB, and the small heat shock chaperones IbpA and IbpB, enhanced the level of soluble and functional recombinant proteins in *E. coli* (Thomas and Baneyx, 1996; Lee et al., 2004; de Marco et al., 2007; Tong et al., 2016; Khosrowabadi et al., 2018). Using a similar approach, soluble cyclohexanone monooxygenase (CHMO) from *Acinetobacter* sp. NCIMB 9871 was successfully expressed in *E. coli* (Lee et al., 2004). CHMO is quite prone to IB formation however, by expressing the protein in concert with either GroEL/GroES or DnaK/DnaJ/GrpE gave a 38-fold improvement. Additionally, expression of foldases DsbA, DsbC, hPPIase with the *CHMO* gene also improved soluble protein expression, though not as high as with molecular chaperones.

Reducing culture temperature represents one strategy for increasing the production of soluble proteins as discussed in a previous section. The slight reduction in temperature has been identified to improve the soluble protein expression slightly (Lee et al., 2004). However, lowering the temperature significantly compromises the ability of chaperones of assisting protein folding in the regular mesophilic *E. coli* cells. To overcome this obstacle, specific strains of *E. coli* that possess cold-adapted chaperone systems have been developed. These strains show improved protein processing abilities at low temperatures. A prime example is the *ArcticExpress* strain, which contains cold-adapted chaperonins of Cpn60 and co-Cpn10 from the psychrophilic bacterium, *Oleispira antarctica* (Ferrer et al., 2003).

#### Expression Systems for Membrane and Toxic Proteins

Membrane proteins overexpression is often met with significant difficulties due to host toxicity and IBs formation. In such cases, the re-solubilization of IBs proteins involves harsh treatments with either detergents (Palmer and Wingfield, 2004), organic solvents, or chaotropes (Jevševar et al., 2005) with limited improvement to the overall protein yield. Engineered *E. coli* strains such as the Walker strains C41 (DE3), C43 (DE3) (Miroux and Walker, 1996), Lemo21 (Wagner et al., 2008), and BL21 (DE3) pLysS are commonly used to produce soluble membrane proteins as well as toxic or particularly challenging proteins. The success of these strains is attributed to their reduced transcriptional activity owing to a variety of beneficial mutations addressed in this section (Figure 2C).

The *E. coli* C41 (DE3) and C43 (DE3) strains (Miroux and Walker, 1996) both possess mutations in the *lac*UV5 promoter and additionally, the C43 (DE3) strain also possesses a mutation in the lac repressor gene (*LacI*). These dampen T7 RNA polymerase expression compared with the wild type *lac* promoter, thus enabling these strains to withstand toxic effects associated with the overexpression of certain membrane proteins (Kwon et al., 2015). By drawing on the same idea, a novel expression system which lowers T7 RNA polymerase expression via repression by a mutant LacI repressor protein (mLacI) was designed (Kim et al., 2017). mLacI possesses the same mutations found in the *LacI* gene in the C43 (DE3) strain which limits its ability to bind inducer molecules such as IPTG. In the mLacI system, expression of this mutant repressor is governed by an L-rhamnose inducible promoter (p*rha*BAD) and fine tuning over transcription can be implemented in a wide range of *lac*O expression systems. The strain *E. coli* BL21 (DE3) pLysS lowers basal level suppression of T7 RNA polymerase with a constitutively expressed T7 lysozyme (carried on the pLysS plasmid). Induction with IPTG then enables the T7 RNA polymerase to overcome this inhibition and carry out transcription of the target protein. Lemo21 (DE3) functions in a similar way, but allows tunable expression of the T7 lysozyme by placing it under the control of the p*rhaBAD* promoter (Wagner et al., 2008).

While these strains enable control over orthogonal T7 RNA polymerases, another means to control protein expression entails the decoupling of recombinant protein expression from host cell growth. The design of this strain, *E. coli* BL21 (DE3):TN7 was inspired by bacteriophage-mediated hijacking of host RNA polymerases to reduce host cell growth, and instead, re-route resources toward the production of viral proteins. The system employs a T7 phage RNA polymerase inhibitor, Gp2, to inhibit endogenous *E. coli* RNA polymerases. Expression of Gp2 is under the control of the p*BAD* promoter and when induced with arabinose, binds to the ß’ jaw domain of *E. coli* RNA polymerase. This results in inhibition of host cell protein production making translation machinery more available for recombinant protein production, thus mitigating inclusion body formation (Stargardt et al., 2020).

Other difficult proteins may also be expressed using *E. coli* SoluB21^TM^ from Genlantis which facilitates soluble expression of particularly difficult mammalian proteins. As a proprietary strain, the mechanism behind soluble protein expression is not well understood. However, certain research groups have had reasonable success with the production of soluble proteins when this strain was used. Hata et al. (2013) purified over 2 mg/L of recombinant human μ-calpain compared with *E. coli* BL21 (DE3) where yields of purified protein was <0.2 mg/L (Hata et al., 2013).

Finally, for industrially relevant biologics, robust *E. coli* expression systems have been designed for scale-up purposes including the *E. coli* pAVEway^TM^ system and more recently, the *E. coli* SoluPro^TM^ systems.

#### Engineering Functional Metal Ion Transport or Biogenic Pathways for Metalloenzymes

For the soluble expression of metalloenzymes, the addition of metal cofactors is a key factor for proper folding and function. While supplementation of metal ions may mitigate inclusion body formation, in certain cases, the overexpression of operons involved in the uptake and transport of specific metal cofactors is required.

Class B Radical S-Adenosylmethionine Methylases (SAM) belong to the family of Radical SAM (RS) enzymes which methylate inactivated carbon and phosphorus centers. This class of RS methylases are cobalamin (Vitamin B12) dependent and possess an iron-sulfur cluster. Attempts to express this protein in *E. coli* have resulted in inclusion bodies, however by overexpressing the cobalamin transport system of *E. coli* (*btuCEDFB* operon) under the control of the pBAD promoter in *E. coli* BL21 (DE3) yielded a significant improvement. In this way, Fom3 from *S. wedmorensis* which produces IBs in *E. coli* achieved 95% soluble expression at a yield of 3 mg/L (Lanz et al., 2018).

Alternatively, strains such as *E. coli* SufFeScient have been engineered to overexpress biogenic pathways (Suf pathway) to produce iron-sulfur cluster containing proteins with full iron occupancy (Corless et al., 2020). Generally, iron-sulfur proteins are re-folded from IBs or expressed as fusion proteins for soluble expression. Utilizing this strain in conjunction with soluble fusion tags may provide an improvement in soluble protein yield, but further research is required to examine the potential benefits of using such a strain to minimize IBs.

### Alternative Protein Expression Hosts to *E. coli*

The numerous strains described here follow a similar theme of modulating the initial burst of transcription associated with strong T7 promoters. However, if the usage of engineered *E. coli*, specifically designed to reduce IBs, is deemed unsuccessful; an alternative host organism may be utilized. Alternative eukaryotic hosts may have the correct PTM machinery, as well as an internal environment that is better suited to express and fold target proteins. Yeast is a popular unicellular host organism that can perform sufficient PTMs with their cell machinery (Malys et al., 2011). Many yeast species have been developed for heterogeneous proteins expression including *Pichia pastoris, Saccharomyces cerevisiae*, and *Kluyveromyces lactis* (Çelik and Çalik, 2012). In addition, insect and mammalian cell lines, transgenic plants, and animals are among the other host systems for recombinant proteins expression systems. Each one of these systems comes with their advantages and disadvantages.

### Construction of Novel Expression Vectors

Introducing certain features to the backbone of used expression vectors may reduce IBs formation and improve the solubility of recombinant proteins. Among these changes are adding a soluble fusion tag to the protein of interest, using weaker promoters to drive protein synthesis, constructing expression vector with low copy number plasmids, and the co-expression of multiple protein components using compatible dual plasmids.

**FIGURE 3:**
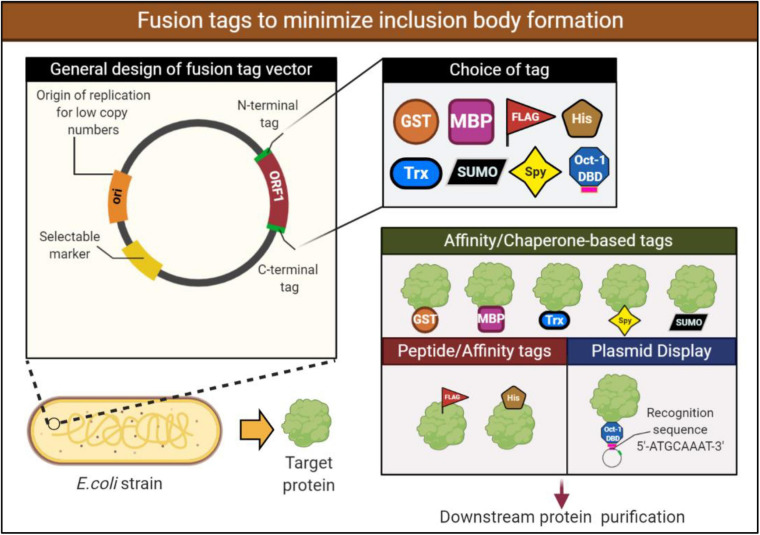
Design of a fusion expression vector to aid in solubilizing the expressed recombinant protein. Expression vectors containing a fusion protein or peptide tags may help increase the solubility of the target protein by promoting proper folding and stabilization. These fusions may be attached to either the N- or C-terminus. Various protein fusions and peptide tags are available for selection, however, they must be carefully chosen to be compatible with the target protein. Examples of protein fusion tags include glutathione-S-transferase (GST), mannose binding protein (MBP), while peptide tags include commonly utilized histidine (His). Plasmid display system technology makes use of fusion partners to attach the fusion and target protein to the plasmid to improve stability of the expressed protein. If selecting a fusion partner to use in a plasmid display system, consideration must be given to an appropriate and soluble fusion partner. For example, in the following figure the transcription factor Oct-1 is utilized which possesses a DBD that recognizes and attaches to the recognition sequence.

Fusing the target protein to a soluble protein or peptide tags can enhance the solubility and reduce IBs levels. There are many fusion tags available for the attachment to either the N or C termini of target proteins. In such cases, the soluble fusion tag helps the expressed fusion protein (fusion tag linked with a target protein) to achieve a better overall solubility. While the mechanism is not well understood, it is thought that fusion to a stable partner assists in stabilizing and promoting proper folding of the insoluble protein. Notably, these fusion tags can be removed from target proteins using the cleavage power of specific proteases working at sites between fusion tags and the target proteins. The widely used fusion partners include glutathione-S-transferase (GST) and maltose-binding protein (MBP). These tags have displayed beneficial effects in improving the solubility of some heterologous proteins, which mainly formed IBs when expressed alone in *E. coli* (Vu et al., 2014; Paraskevopoulou and Falcone, 2018). Examples of other popular tags used in enhancing recombinant protein solubility include the small ubiquitin-like modifier (SUMO), thioredoxin (Trx), and N-utilization substance A (NusA) tags. An alternative to protein fusion tags are small peptide tags such as the commonly employed His tag. While peptide tags employ much of the similar benefits to protein fusions, peptide tags are smaller, generally up to 15 amino acids. The benefit of a smaller tag is that they are less likely to interfere with the structure and potentially function of the target protein (Paraskevopoulou and Falcone, 2018). Not all fusion tags will work efficiently with any protein, therefore, the fusion must be chosen to be compatible with the protein of interest (Figure 3).

Fusion of the target protein directly to molecular chaperones themselves presents an interesting alternative of utilizing the ability of chaperones through a fusion system. This interaction allows for a more facilitated interaction between the protein and chaperone leading to the mediated folding via the chaperone and therefore preventing formation of the inclusion bodies (Costa et al., 2014). Fusions utilizing chaperones have been validated in the past (Kyratsous et al., 2009). This methodology was brought back to attention recently using spheroplast protein Y (Spy) (Ruan et al., 2020). Spy is a periplasmic chaperone found in bacteria that inhibits aggregation independent of ATP and other chaperones (Evans et al., 2011; Quan et al., 2011). Spy was fused to the N terminus of different mammalian proteins in the experiment and was able to retain its chaperone activity. Furthermore, a clear improvement to the solubility of the proteins was observed vs. the non-fused proteins (Ruan et al., 2020). These recent advancement in utilizing the Spy chaperone highlighted the potential of this method and will likely propel this research and application forward.

Plasmid display system are another approach on fusions proteins wherein the proteins expressed are directly attached to the plasmid. The target protein is expressed *in vivo* and binds to a specific DNA sequence on the encoding plasmid via the DNA binding domain (DBD) that is fused to the target protein (Choi et al., 2005). Selection of the DNA-binding protein is critical to the success of the display system. The DNA-binding protein must be soluble when expressed in *E. coli* but must also possess a high binding affinity (Kd) with the recognition sequence on the plasmid to be effective. Although numerous display systems exist such as phage display (Smith, 1985), cell display (Chen et al., 2001), ribosome display (Mattheakis et al., 1994) and mRNA display (Roberts and Szostak, 1997), the stability of DNA allows for more variable conditions during downstream processes. The use of plasmid display technology has been validated in the past and is picking up more traction in recent times (Choi et al., 2005; Park et al., 2013; Figure 3). Park et al. utilized the Oct-1’s DBD fused to target proteins for the purposes of screening engineered proteins (Park et al., 2013). One of the protein, an antibody fragment (M18 scFv) requires disulfide bonds for functional activity, despite this, efficient expression of M18 scFv was observed utilizing this technology within the *E. coli* SHuffle T7 express lysY strain (Park et al., 2013). Heterologous enzymes expressed within *E. coli* may aggregate or be unstable, however, being bound to a stable molecule such as DNA may improve their stability and reduce levels of aggregation. More recently, Park et al. was involved in the stable expression of immobilized enzymes within *E. coli* using the plasmid display method (Park et al., 2020). Once again the Oct-1 DBD was fused to two fucosyltransferases, and soluble expression and functional activity was improved in the fused constructs (Park et al., 2020). Further research into this technology tackling the issue of inclusion bodies is needed to optimize and provide further proof of concept. However, the technology presents potential as a novel and alternative application in preventing IB formation and may be of further interest in the field.

Besides reducing protein synthesis rates (“Host Engineering for Recombinant Protein Expression in Soluble Form” section), the use of weak promoters and/or a low copy number plasmid is considered another distinctive approach to achieve the above goal. T7 is a popular and strong promoter for protein expression in *E. coli*. Alternative moderately strong or weak promoters may be beneficial to express recombinant proteins that are prone to inclusion bodies formation. Examples of such promoters for this purpose include the *tac*, *araC*, and synthetic *trc* promoters (Lebendiker and Danieli, 2014; Kaur et al., 2018).

Similarly and while high-copy number plasmids are capable of providing the host with many functions that are important for recombinant proteins expression/production including the screening/cloning of genes of interest through selection markers utilization (drug or antibiotic resistance), such plasmids can increase the metabolic burden of the host. To minimize such effects in *E. coli* cells, plasmids are engineered to control their replication with a defined copy number (Del Solar and Espinosa, 2002). A high copy number generally corresponds to 100 copies/cell, while a low copy number is anywhere from 0 to 50 copies/cell. High copy number expression plasmids can lead to inclusion bodies formation due to the high rate of heterogeneous protein expression, thus a low copy number plasmid is more beneficial to yield soluble proteins (Singh et al., 2015).

The co-expression of proteins using dual vectors has the potential to achieve soluble, and active protein complexes while protecting individual subunits from degradation. This is true as the expression of separate components of protein complexes individually often results in IBs formation. For example, the co-expression of *bphI* and *bphJ* in *E. coli* using two compatible plasmids (e.g., pBTL4 and pET28a) yielded a soluble and functional BphI-BphJ complex (Baker et al., 2009). Dual vectors, driven by *T7* promoters, are designed to co-express two (up to eight) target proteins in *E. coli*, which allow host strains to simultaneously express any targeted proteins/chaperone combinations (Tolia and Joshua-Tor, 2006).

### Modifying the Protein of Interest

Given the tight correlation between certain properties of proteins and their propensity to form IBs as discussed in “The Mechanisms and the Influencing Factors of Protein Inclusion Bodies Formation in *E. coli*” section, several bioinformatics tools have been developed to predict protein solubility such as Protein-Sol (Hebditch et al., 2017) and SoDoPE (Bhandari et al., 2020) or identify sequence signatures that lead to protein aggregation, including PASTA 2.0, AMYLPRED 2, Aggrescan, and more others (Conchillo-Solé et al., 2007; Tsolis et al., 2013; Walsh et al., 2014). These tools can be used to minimize protein IBs formation through identifying and then modifying such sequence signatures through site-specific mutagenesis. Such an approach was exemplified by a native *Candida Antarctica* lipase B (CAL-B) that is prone to form IBs in *E. coli* (Jung and Park, 2008). After replacing five hydrophobic residues (Leu147, Leu199, Leu219, Leu261, and Ileu255) with aspartate on the surface of CAL-B, the mutated CAL-B displayed a substantial increased activity and yield in comparison with the wild type enzyme (Jung and Park, 2008). Furthermore, the expression of soluble and truncated domains of the desired recombinant protein can aid in the production of soluble yet functional proteins (Yumerefendi et al., 2010). However, these two approaches may not be a viable solution in case modifying targeted protein sequences impairs their functions.

While the inability to form disulfide bonds within the cytoplasmic space of *E. coli* might be addressed by engineering *trxB*^–^ and *gor*^–^ mutants as mentioned earlier, an alternative solution is through expressing proteins within the periplasmic space that supports the proper disulfide bonding through its oxidative environment and the presence of foldases (including DsbA and DsbC) (Manta et al., 2019). Linking specific signal peptide to a target protein will localize the target protein to the periplasm via the secretory dependent pathway (Sec), signal recognition particle pathway, or twin-arginine translocation pathway (Tat). The prokaryotic signal peptide sequences OmpA and PhoA are often used for this purpose (Humphreys et al., 2000). The pelB signal sequence from *Pectobacterium carotovorum* was successfully used to secrete mouse scFv 13R4 antibody fragment into the periplasmic space (Selas Castiñeiras et al., 2018). Among the additional advantage of expressing proteins in the periplasmic space is their protection against proteolytic cleavages, which can also be obtained by secretory expression outside the outer membrane as mentioned earlier (“Optimized Glycoengineered *E. coli* Strains for N-Linked Glycosylation” section). This is often desirable to obtain higher yields of the target protein/enzymes as they are not subject to proteases (Faizal et al., 2006).

## Potential Advantages of Protein Inclusion Bodies in Industrial Applications

IBs have unique characteristics that may be exploited for medical research and biotechnological applications. First, they are mechanically and chemically stable which constitutes the foundation for their emerging applications as a biomaterial in biomedicine (Rinas et al., 2017; De Marco et al., 2019). Second, the propensity of IBs to aggregate homogenously with a high density presents an opportunity for quick isolation of expressed proteins (Ramón et al., 2014). Moreover and in certain cases, research has demonstrated that inclusion bodies formed by certain proteins might contain bio-functional preparations (Singh et al., 2020; Singhvi et al., 2020) despite the earlier common notion of mostly un-functional and inactive protein complexes within purified IBs. Such promising observations can be further investigated for the factors that affect such functionalities and decipher the mechanism(s) behind the observed differences among individual proteins.

Industrial applications of protein IBs have gained momentum with the current technological advancements and research efforts invested in recent years. Lately, IBs have been used in establishing protein scaffolds through tissue engineering to stimulate cell proliferation and enhance cell attachment through adhesion (Seras-Franzoso et al., 2012; Loo et al., 2015), immobilizing enzymes and the use of cascade enzymatic reactions for enhanced formation of products (Han et al., 2017), and serving as drug-delivery systems (Liovic et al., 2012). Additionally, IBs formed from therapeutic proteins (e.g., Hsp70, catalase, dihydrofolate reductase, and leukemia inhibitory factor) have been shown to increase the viability of target cells placed under certain stress conditions when such IBs are added to the culture media (Vázquez et al., 2012). Moreover, the use of IBs, as a controlled protein packaging and delivery system, was demonstrated to contribute to the partial reconstruction of cytoskeleton through the utilization of keratin inclusion bodies (Liovic et al., 2012). Catalytically active IBs have also been used to synthesize key precursors of important pharmaceutical drugs. For example, sialic acid aldolase (SAA) is used in industrial settings to produce neuraminic acid, which is the precursor of the antivirotic drug Relenza^®^. In this case, an N-terminal cellulose-binding domain from *Clostridium cellulovorans* with self-aggregation was fused to the SAA gene and expressed in *E. coli* BL21 (DE3) cells forming catalytically active IBs. These IBs showed the same activity as the soluble SAA enzyme while the lyophilized catalytic IBs showed 93% of the original activity (Nahálka et al., 2008). Significant strides have also been made toward boosting *E. coli* IBs formation of other pharmaceutically relevant drugs using continuous fed-batch cultivation (Slouka et al., 2019). For example, catalytically active inclusion bodies were engineered most recently to produce 1,5-diaminopentane (DAP), a natural polyamine with broad prospects for various applications/bio-based polyamides/bioactivities (Ma et al., 2017). By using catalytically active IBs of the constitutive L-lysine decarboxylase (expressed in *E. coli*) to process L-lysine-containing culture supernatants from *Corynebacterium glutamicum*, high conversions to DAP (87–100%) were obtained in 30–60 mL batch reactions (Kloss et al., 2018).

Similarly, an industrial lysozyme of SLLyz from the insect *Spodoptera litura* was produced as IBs containing a 121 amino acids polypeptide fused at the C-terminal to GST. After purification, this lysozyme demonstrated strong antibacterial activity against *Bacillus megaterium*, providing a dependable approach for maximizing production and purification of such important recombinant polypeptides (Kim et al., 2011). Moreover, IBs proved to be a pivotal tool for the expression and purification of numerous antimicrobial peptides that are of commercial importance. The antibacterial, antiviral, antifungal, and antiparasitic properties of such short (10–100 amino acids) positively charged polypeptides makes them a very interesting target for commercial implementations, especially in light of the current unprecedented spread of multi-drug resistant microbes. The decreased solubility, small-size, susceptibility to degradation, and elevated host-toxicity of such peptides makes the use of IBs heterologous expression the most efficient approach to manage production costs, as well as masking host toxicity issues and protecting the expressed peptides from proteolytic cleavage (Köszagová and Nahálka, 2020).

It should be noted however that *E. coli* IBs of therapeutic proteins can trigger in some cases endotoxic immune responses in humans due to the presence of lipopolysaccharides. Cases such as these have revived interest in the use of gram-positive strains such as food-grade *Lactococcus lactis* for the production of functional IBs (Cano-Garrido et al., 2016). Notably, there are commercially available strains of *E. coli* such as ClearColi^TM^ that do not produce LPS (Mamat et al., 2013).

Finally, a good example for IBs potential industrial usages which was recently explored is the use of IBs in the production of 1-Butanol. This solvent is commonly utilized in many industries including but not limited to flavorings, cosmetics, and brake fluids. In addition to its use in repellants production, it is also considered an essential factor in the manufacturing of dietary vitamins and vegetable oils as well as antibiotics and hormones (Green, 2011). The natural presence of 1-Butanol in alcoholic beverages, chesses, fruits, and a variety of other foods (as a by-product of carbohydrate fermentation), negates any concerns to its safety within the reported concentrations (Macholz, 1989). In the above explored application, IBs were designed to harbor heterologous enzymes that are involved in 1-Butanol production alongside a carbon binding domain (CBD) (Han et al., 2017). The enzymes and the CBD interacted through a leucine zipper motive mimicking a prey-bait system to achieve active IBs for 1-Butanol production, leading to a 1.5-fold increase in 1-Butanol yields compared to the control (García-Fruitõs et al., 2011; Villaverde et al., 2015).

## Discussion and Outlook

*E. coli* is a popular microbial host for protein expression, where recombinant proteins applications often encounter issues with IBs formation. Many strategies have been developed to reduce the formation of IBs in *E. coli*, involving the fine-tuning of protein expression rates, engineering host strains to enable key post-translational modifications, tuning the expression vector appropriately, and using bioinformatics tools to predict the tendency of proteins to aggregate. These strategies were discussed extensively for the aims to reduce and prevent IBs formation. Furthermore, the review discussed specific methods and examples to achieve the desired goals of many industrial applications while addressing any shortcomings. The future better understanding in the regulation mechanism of protein homeostasis network will certainly facilitate the development of strategies to minimize protein IBs formation in *E. coli*. In addition, the recently changing views on classical IBs may be attributed to the discovery of functionally active IBs. These changing views have enabled the breakthrough of IBs into industrial areas for the production of compounds, and future use in medicines. While the field agreeably is at its infancy with a limited number of reports/experiments, research endeavors focusing on IBs within the large industrial scale are encouraged in order to refine future applications, better understand the various aspects of *E. coli* IBs, and identify mitigation strategies.

## Author Contributions

AB and WW were involved in the development of the topic and initial drafts. YH and NA contributed by including and writing further relevant ideas of discussion to the topic. X-ZL and all other listed authors assisted in editing and peer-reviewing the manuscript. TZ conceived and coordinated the study. All authors contributed to the article and approved the submitted version.

## Conflict of Interest

The authors declare that the research was conducted in the absence of any commercial or financial relationships that could be construed as a potential conflict of interest.

## References

[B1] Baker P. Pan D. Carere J. Rossi A. Wang W. Seah S. Y. K. (2009). Characterization of an aldolase-dehydrogenase complex that exhibits substrate channeling in the polychlorinated biphenyls degradation pathway. *Biochemical* 48 6551–6558. 10.1021/bi9006644 19476337

[B2] Balch W. E. Morimoto R. I. Dillin A. Kelly J. W. (2008). Adapting proteostasis for disease intervention. *Science* 319 916–919. 10.1126/science.1141448 18276881

[B3] Baumgarten T. Ytterberg A. J. Zubarev R. A. De Gier J. -W. (2018). Optimizing recombinant protein production in the *Escherichia coli* periplasm alleviates stress. *Appl. Environ. Microbiol.* 84:e00270-18 10.1128/AEM.00270-18 PMC5981079 29654183

[B4] Bessette P. H. Åslund F. Beckwith J. Georgiou G. (1999). Efficient folding of proteins with multiple disulfide bonds in the *Escherichia coli* cytoplasm. *Proc. Natl. Acad. Sci. U. S. A.* 96 13703–13708. 10.1073/pnas.96.24.13703 10570136 PMC24128

[B5] Bhandari B. K. Gardner P. P. Lim C. S. (2020). Solubility-weighted index: fast and accurate prediction of protein solubility. *Bioinformatics* 36 4691–4698. 10.1093/bioinformatics/btaa578 32559287 PMC7750957

[B6] Biancalana M. Koide S. (2010). Molecular mechanism of thioflavin-T binding to amyloid fibrils. *Biochim. Biophys. Acta* 1804 1405–1412. 10.1016/j.bbapap.2010.04.001 20399286 PMC2880406

[B7] Blackwell J. R. Horgan R. (1991). A novel strategy for production of a highly expressed recombinant protein in an active form. *FEBS Lett.* 295 10–12 10.1016/0014-5793(91)81372-f 1765138

[B8] Blakemore F. (1947). Conjunctivitis and keratitis of cattle and sheep associated with the presence of cell-inclusion bodies. *J. Comp. Pathol. Ther.* 57 223–231 10.1016/s0368-1742(47)80028-1 20265766

[B9] Bothmann H. Plückthun A. (2000). The periplasmic *Escherichia coli* peptidylprolyl cis,trans-isomerase FkpA: i. increased functional expression of antibody fragments with and without cis-prolines. *J. Biol. Chem.* 275 17100–17105. 10.1074/jbc.m910233199 10748200

[B10] Bowden G. A. Paredes A. M. Georgiou G. (1991). Structure and morphology of protein inclusion bodies in *E.coli*. *Nat. Biotechnol.* 9 725–730. 10.1038/nbt0891-725 1367632

[B11] Browning D. F. Godfrey R. E. Richards K. L. Robinson C. Busby S. J. W. (2019). Exploitation of the *Escherichia coli* lac operon promoter for controlled recombinant protein production. *Biochem. Soc. Trans.* 47 755–763. 10.1042/bst20190059 30971435

[B12] Bushmarina N. A. Blanchet C. E. Vernier G. Forge V. (2006). Cofactor effects on the protein folding reaction: acceleration of alpha-lactalbumin refolding by metal ions. *Protein Sci.* 15 659–671. 10.1110/ps.051904206 16522796 PMC2242491

[B13] Cabilly S. (1989). Growth at sub-optimal temperatures allows the production of functional, antigen-binding Fab frag-ments in *Escherichia coli*. *Gene* 89 553–557. 10.1016/0378-1119(89)90451-4 2697647

[B14] Cano-Garrido O. Sánchez-Chardi A. Parés S. Giró I. Tatkiewicz W. I. Ferrer-Miralles N. (2016). Functional protein-based nanomaterial produced in microorganisms recognized as safe: a new platform for biotechnology. *Acta Biomater.* 43 230–239. 10.1016/j.actbio.2016.07.038 27452157

[B15] Carere J. Hassan Y. I. Lepp D. Zhou T. (2018a). The enzymatic detoxification of the mycotoxin deoxynivalenol: identification of DepA from the DON epimerization pathway. *Microb. Biotechnol.* 11 1106–1111. 10.1111/1751-7915.12874 29148251 PMC6196400

[B16] Carere J. Hassan Y. I. Lepp D. Zhou T. (2018b). The identification of DepB: an enzyme responsible for the final detoxification step in the deoxynivalenol epimerization pathway in devosia mutans 17-2-E-8. *Front. Microbiol.* 9:1573. 10.3389/fmicb.2018.01573 PMC6056672 30065709

[B17] Carriö M. M. Cubarsi R. Villaverde A. (2000). Fine architecture of bacterial inclusion bodies. *FEBS Lett.* 471 7–11. 10.1016/s0014-5793(00)01357-0 10760503

[B18] Carrió M. González-Montalbán N. Vera A. Villaverde A. Ventura S. (2005). Amyloid-like properties of bacterial inclusion bodies. *J. Mol. Biol.* 347 1025–1037. 10.1016/j.jmb.2005.02.030 15784261

[B19] Castellanos-Mendoza A. Castro-Acosta R. M. Olvera A. Zavala G. Mendoza-Vera M. García-Hernández E. (2014). Influence of pH control in the formation of inclusion bodies during production of recombinant sphingomyelinase-D in *Escherichia coli*. *Microb. Cell Fact.* 13:137. 10.1186/s12934-014-0137-9 PMC4177172 25213001

[B20] Çelik E. Çalik P. (2012). Production of recombinant proteins by yeast cells. *Biotechnol. Adv.* 30 1108–1118. 10.1016/j.biotechadv.2011.09.011 21964262

[B21] Chapman M. R. Robinson L. S. Pinkner J. S. Roth R. Heuser J. Hammar M. (2002). Role of *Escherichia coli* curli operons in directing amyloid fiber formation. *Science* 295 851–855. 10.1126/science.1067484 11823641 PMC2838482

[B22] Chen G. Hayhurst A. Thomas J. G. Harvey B. R. Iverson B. L. Georgiou G. (2001). Isolation of high-affinity ligand-binding proteins by periplasmic expression with cytometric screening (PECS). *Nat. Biotechnol.* 19 537–542. 10.1038/89281 11385457

[B23] Chen J. Yu J. Tang L. Tang M. Shi Y. Pang Y. (2003). Comparison of the expression of Bacillus thuringiensis full-length and N-terminally truncated vip3A gene in *Escherichia coli*. *J. Appl. Microbiol.* 95 310–316. 10.1046/j.1365-2672.2003.01977.x 12859763

[B24] Chen R. (2012). Bacterial expression systems for recombinant protein production: *E. coli* and beyond. *Biotechnol. Adv.* 30 1102–1107. 10.1016/j.biotechadv.2011.09.013 21968145

[B25] Choi J. H. Lee S. Y. (2004). Secretory and extracellular production of recombinant proteins using *Escherichia coli*. *Appl. Microbiol. Biotechnol.* 64 625–635. 10.1007/s00253-004-1559-9 14966662

[B26] Choi J. H. Jeong K. J. Kim S. C. Lee S. Y. (2000). Effcient secretory production of alkaline phosphatase by high cell density culture of recombinant *Escherichia coli* using the Bacillus sp. endoxylanase signal sequence. *Appl. Microbiol. Biotechnol.* 53 640–645. 10.1007/s002530000334 10919319

[B27] Choi Y. S. Pack S. P. Yoo Y. J. (2005). Development of a plasmid display system using GAL4 DNA binding domain for the in vitro screening of functional proteins. *Biotechnol. Lett.* 27 1707–1711. 10.1007/s10529-005-2735-4 16247679

[B28] Clark D. J. Maaløe O. (1967). DNA replication and the division cycle in *Escherichia coli*. *J. Mol. Biol.* 23 99–112. 10.1016/s0022-2836(67)80070-6

[B29] Conchillo-Solé O. de Groot N. S. Avilés F. X. Vendrell J. Daura X. Ventura S. (2007). AGGRESCAN: a server for the prediction and evaluation of “hot spots” of aggregation in polypeptides. *BMC Bioinformatics* 8:65. 10.1186/1471-2105-8-65 17324296 PMC1828741

[B30] Corless E. I. Mettert E. L. Kiley P. J. Antony E. (2020). Elevated expression of a functional suf pathway in *Escherichia coli* BL21(DE3) enhances recombinant production of an iron-sulfur cluster-containing protein. *J. Bacteriol.* 202 1–11. 10.1128/JB.00496-19 PMC6964742 31712282

[B31] Costa S. Almeida A. Castro A. Domingues L. (2014). Fusion tags for protein solubility, purification, and immunogenicity in *Escherichia coli*: the novel Fh8 system. *Front. Microbiol.* 5:63. 10.3389/fmicb.2014.00063 PMC3928792 24600443

[B32] de Groot N. S. Ventura S. (2006). Effect of temperature on protein quality in bacterial inclusion bodies. *FEBS Lett.* 580 6471–6476. 10.1016/j.febslet.2006.10.071 17101131

[B33] de Marco A. Deuerling E. Mogk A. Tomoyasu T. Bukau B. (2007). Chaperone-based procedure to increase yields of soluble recombinant proteins produced in *E. coli*. *BMC Biotechnol.* 7:32. 10.1186/1472-6750-7-32 17565681 PMC1904446

[B34] De Marco A. Ferrer-Miralles N. Garcia-Fruitós E. Mitraki A. Peternel S. Rinas U. (2019). Bacterial inclusion bodies are industrially exploitable amyloids. *FEMS Microbiol. Rev.* 43 53–72. 10.1093/femsre/fuy038 30357330

[B35] De Strooper B. Karran E. (2016). The cellular phase of Alzheimer’s disease. *Cell* 164 603–615. 26871627 10.1016/j.cell.2015.12.056

[B36] Del Solar G. Espinosa M. (2002). Plasmid copy number control: an ever-growing story. *Mol. Microbiol.* 37 492–500. 10.1046/j.1365-2958.2000.02005.x 10931343

[B37] Diamant S. Eliahu N. Rosenthal D. Goloubinoff P. (2001). Chemical chaperones regulate molecular chaperones in vitro and in cells under combined salt and heat stresses. *J. Biol. Chem.* 276 39586–39591. 10.1074/jbc.m103081200 11517217

[B38] Ding N. Fu X. Ruan Y. Zhu J. Guo P. Han L. (2019). Extracellular production of recombinant N-glycosylated anti-VEGFR2 monobody in leaky *Escherichia coli* strain. *Biotechnol. Lett.* 41 1265–1274. 10.1007/s10529-019-02731-0 31541332

[B39] Donovan R. S. Robinson C. W. Glick B. R. (1996). Review: optimizing inducer and culture conditions for expression of foreign proteins under the control of the lac promoter. *J Industr. Microbiol.* 16 145–154. 10.1007/bf01569997 8652113

[B40] Dow B. A. Tatulian S. A. Davidson V. L. (2015). Use of the amicyanin signal sequence for efficient periplasmic expression in *E. coli* of a human antibody light chain variable domain. *Protein Expr. Purif.* 108 9–12. 10.1016/j.pep.2014.12.017 25573388 PMC4363176

[B41] Dyson M. R. Shadbolt S. P. Vincent K. J. Perera R. L. McCafferty J. (2004). Production of soluble mammalian proteins in *Escherichia coli*: identification of protein features that correlate with successful expression. *BMC Biotechnol.* 4:32. 10.1186/1472-6750-4-32 PMC544853 15598350

[B42] Espinoza A. M. Medina V. Hull R. Markham P. G. (1991). Cauliflower mosaic virus gene II product forms distinct inclusion bodies in infected plant cells. *Virol.* 185 337–344. 10.1016/0042-6822(91)90781-6 1656590

[B43] Evans M. L. Schmidt J. C. Ilbert M. Doyle S. M. Quan S. Bardwell J. C. A. (2011). *E. coli* chaperones DnaK, Hsp33 and Spy inhibit bacterial functional amyloid assembly. *Prion* 5 323–334. 10.4161/pri.5.4.18555 22156728 PMC3821533

[B44] Faizal A. Razis A. Nur E. Ismail B. Hambali Z. Nazrul M. (2006). The periplasmic expression of recombinant human epidermal growth factor (hEGF) in *Escherichia coli*. *Asia Pacific J. Mol. Biol. Biotechnol.* 14 249–261.

[B45] Feldman M. F. Wacker M. Hernandez M. Hitchen P. G. Marolda C. L. Kowarik M. (2005). Engineering N-linked protein glycosylation with diverse O antigen lipopolysaccharide structures in *Escherichia coli*. *PNAS* 102 3016–3021. 10.1073/pnas.0500044102 15703289 PMC549450

[B46] Ferrer M. Chernikova T. N. Yakinov M. M. Golyshin P. N. Timmis K. N. (2003). Chaperonins govern growth of *Escherichia coli* at low temperatures. *Nat. Biotechnol.* 21 1266–1267. 10.1038/nbt1103-1266 14595348

[B47] Francis M. D. Page R. (2010). Strategies for optimizing heterologous protein expression in *Escherichia coli*. *Curr. Protoc. Protein Sci.* 5 1–29. 10.1016/j.pep.2006.06.024 16904906

[B48] Gaciarz A. Khatri N. K. Velez-Suberbie M. L. Saaranen M. J. Uchida Y. Keshavarz-Moore E. (2017). Efficient soluble expression of disulfide bonded proteins in the cytoplasm of *Escherichia coli* in fed-batch fermentations on chemically defined minimal media. *Microb. Cell Fact.* 16:108. 10.1186/s12934-017-0721-x PMC5471842 28619018

[B49] García-Fruitõs E. Sabate R. De Groot N. S. Villaverde A. Ventura S. (2011). Biological role of bacterial inclusion bodies: a model for amyloid aggregation. *FEBS J.* 278 2419–2427. 10.1111/j.1742-4658.2011.08165.x 21569209

[B50] Georgalis Y. Starikov E. B. Hollenbach B. Lurz R. Scherzinger E. Saenger W. (1998). Huntingtin aggregation monitored by dynamic light scattering. *Proc. Natl. Acad. Sci. U. S. A.* 95 6118–6121. 10.1073/pnas.95.11.6118 9600927 PMC27595

[B51] Gill R. T. Valdes J. J. Bentley W. E. (2000). A comparative study of global stress gene regulation in response to overexpression of recombinant proteins in *Escherichia coli*. *Metab. Eng.* 2 178–189 10.1006/mben.2000.0148 11056060

[B52] Goh C. S. Lan N. Douglas S. M. Wu B. Echols N. Smith A. (2004). Mining the structural genomics pipeline: identification of protein properties that affect high-throughput experimental analysis. *J. Mol. Biol.* 336 115–130. 10.1016/j.jmb.2003.11.053 14741208

[B53] Gomes R. A. Byregowda S. M. Veeregowda M. Balamurugan V. (2016). An overview of heterologous expression host systems for the production of recombinant proteins. *Adv. Anim. Vet. Sci.* 4 346–356. 10.14737/journal.aavs/2016/4.7.346.356

[B54] Green E. M. (2011). Fermentative production of butanol-the industrial perspective. *Curr. Opin. Biotechnol.* 22 337–343. 10.1016/j.copbio.2011.02.004 21367598

[B55] Gregersen N. Bross P. Vang S. Christensen J. H. (2006). Protein misfolding and human disease. *Annu. Rev. Genomics Hum. Genet.* 7 103–124. 16722804 10.1146/annurev.genom.7.080505.115737

[B56] Grossman T. Kawasaki E. Punreddy S. Osburne M. (1998). Spontaenous cAMP-dependent derepression of gene expression. *Gene* 209 95–103. 10.1016/s0378-1119(98)00020-1 9524234

[B57] Han G. H. Seong W. Fu Y. Yoon P. K. Kim S. K. Yeom S. J. (2017). Leucine zipper-mediated targeting of multi-enzyme cascade reactions to inclusion bodies in *Escherichia coli* for enhanced production of 1-butanol. *Metab. Eng.* 40 41–49. 10.1016/j.ymben.2016.12.012 28038953

[B58] Hassan Y. Zhu H. Zhu Y. Zhou T. (2016). Beyond ribosomal binding: the increased polarity and aberrant molecular interactions of 3-epi-deoxynivalenol. *Toxins* 8:261. 10.3390/toxins8090261 27618101 PMC5037487

[B59] Hata S. Kitamura F. Sorimachi H. (2013). Efficient expression and purification of recombinant human μ-calpain using an *Escherichia coli* expression system. *Genes Cells* 18 753–763. 10.1111/gtc.12071 23786391

[B60] Hatahet F. Ruddock L. W. (2013). Topological plasticity of enzymes involved in disulfide bond formation allows catalysis in either the periplasm or the cytoplasm. *J. Mol. Biol.* 425 3268–3276. 10.1016/j.jmb.2013.04.034 23810903

[B61] Hebditch M. Carballo-Amador M. A. Charonis S. Curtis R. Warwicker J. (2017). Protein-sol: a web tool for predicting protein solubility from sequence. *Bioinformatics* 33, 3098–3100. 10.1093/bioinformatics/btx345 28575391 PMC5870856

[B62] Hoffmann F. Rinas U. (2000). Kinetics of heat-shock response and inclusion body formation during temperature-induced production of basic fibroblast growth factor in high-cell-density cultures of recombinant *Escherichia coli*. *Biotechnol. Prog.* 16 1000–1007. 10.1021/bp0000959 11101327

[B63] Humphreys D. P. Sehdev M. Chapman A. P. Ganesh R. Smith B. J. King L. M. (2000). High-level periplasmic expression in *Escherichia coli* using a eukaryotic signal peptide: importance of codon usage at the 5’ end of the coding sequence. *Protein Expr. Purif.* 20 252–264. 10.1006/prep.2000.1286 11049749

[B64] Jevševar S. Gaberc-Porekar V. Fonda I. Podobnik B. Grdadolnik J. Menart V. (2005). Production of nonclassical inclusion bodies from which correctly folded protein can be extracted. *Biotechnol. Prog.* 21 632–639. 10.1021/bp0497839 15801811

[B65] Jhamb K. Sahoo D. K. (2012). Production of soluble recombinant proteins in *Escherichia coli*: effects of process conditions and chaperone co-expression on cell growth and production of xylanase. *Bioresour. Technol.* 123 135–143. 10.1016/j.biortech.2012.07.011 22940310

[B66] Jimenez-Sanchez M. Licitra F. Underwood B. R. Rubinsztein D. C. (2017). Huntington’s disease: mechanisms of pathogenesis and therapeutic strategies. *Cold Spring Harb. Perspect. Med.* 7:a024240. 10.1101/cshperspect.a024240 PMC5495055 27940602

[B67] Jordal P. B. Dueholm M. S. Larsen P. Petersen S. V. Enghild J. J. Christiansen G. (2009). Widespread abundance of functional bacterial amyloid in mycolata and other gram-positive bacteria. *Appl. Environ. Microbiol.* 75 4101–4110. 10.1128/aem.02107-08 19395568 PMC2698375

[B68] Jung S. Park S. (2008). Improving the expression yield of *Candida antarctica* lipase B in *Escherichia coli* by mutagenesis. *Biotechnol. Lett.* 30 717–722. 10.1007/s10529-007-9591-3 17985077

[B69] Jung S. Koo B. K. Chong S. H. Kim K. Choi D. K. Vu T. T. T. (2013). Soluble expression of human leukemia inhibitory factor with protein disulfide isomerase in *Escherichia coli* and its simple purification. *PLoS One* 8:e83781. 10.1371/journal.pone.0083781 24358310 PMC3865251

[B70] Kalia L. V. Lang A. E. (2015). Parkinson’s disease. *Lancet* 386 896–912. 25904081 10.1016/S0140-6736(14)61393-3

[B71] Katoh Y. Y. Yamazaki E. Taniguti K. Yamada K. Isomura G. (2006). Light and electron microscopic observation of intracytoplasmic inclusion bodies in the locus Coerleus of the hamstern the locus coeruleus of hamster. *Arch. Histol. Cytol.* 69 129–134. 10.1679/aohc.69.129 16819152

[B72] Kaur J. Kumar A. Kaur J. (2018). Strategies for optimization of heterologous protein expression in *E. coli*: roadblocks and reinforcements. *Int. J. Biol. Macromol.* 106 803–822. 10.1016/j.ijbiomac.2017.08.080 28830778

[B73] Khosrowabadi E. Takalloo Z. Sajedi R. H. Khajeh K. (2018). Improving the soluble expression of aequorin in *Escherichia coli* using the chaperone-based approach by co-expression with artemin. *Prep. Biochem. Biotechnol.* 48 483–489. 10.1080/10826068.2018.1466152 29958068

[B74] Kikkawa Y. Spitzer R. (1969). Inclusion bodies of type II alveolar cells: species differences and morphogenesis. *Anat. Rec.* 163 525–541. 10.1002/ar.1091630405 5776880

[B75] Kim J. W. Yoe J. Lee G. H. Yoe S. M. (2011). Recombinant expression and refolding of the c-type lysozyme from *Spodoptera litura* in *E. coli*. *Electron. J. Biotechnol.* 14:6

[B76] Kim S. K. Lee D. H. Kim O. C. Kim J. F. Yoon S. H. (2017). Tunable control of an *Escherichia coli* expression system for the overproduction of membrane proteins by titrated expression of a mutant lac repressor. *ACS Synth. Biol.* 6 1766–1773. 10.1021/acssynbio.7b00102 28524655

[B77] Kim Y. E. Hipp M. S. Bracher A. Hayer-Hartl M. Ulrich Hartl F. (2013). Molecular chaperone functions in protein folding and proteostasis. *Annu. Rev. Biochem.* 82 323–355. 10.1146/annurev-biochem-060208-092442 23746257

[B78] Kloss R. Limberg M. H. Mackfeld U. Hahn D. Grünberger A. Jäger V. D. (2018). Catalytically active inclusion bodies of L-lysine decarboxylase from *E. coli* for 1,5-diaminopentane production. *Sci. Rep.* 8:5856. 10.1038/s41598-018-24070-2 PMC5895699 29643457

[B79] Köszagová R. Nahálka J. (2020). Inclusion bodies in biotechnology. *J. Microbiol. Biotechnol. Food Sci.* 9 1191–1196. 10.15414/jmbfs.2020.9.6.1191-1196

[B80] Krachmarova E. Ivanov I. Nacheva G. (2020). Nucleic acids in inclusion bodies obtained from *E. coli* cells expressing human interferon-gamma. *Microb. Cell Fact.* 19:139. 10.1186/s12934-020-01400-6 PMC7353671 32652996

[B81] Kusano K. Waterman M. R. Sakaguchi M. Omura T. Kagawa N. (1999). Protein synthesis inhibitors and ethanol selectively enhance heterologous expression of P450s and related proteins in *Escherichia coli*. *Arch. Biochem. Biophys.* 367 129–136 10.1006/abbi.1999.1248 10375408

[B82] Kwon S. K. Kim S. K. Lee D. H. Kim J. F. (2015). Comparative genomics and experimental evolution of *Escherichia coli* BL21(DE3) strains reveal the landscape of toxicity escape from membrane protein overproduction. *Sci. Rep.* 5:16076. 10.1038/srep16076 PMC4632034 26531007

[B83] Kyratsous C. A. Silverstein S. J. DeLong C. R. Panagiotidis C. A. (2009). Chaperone-fusion expression plasmid vectors for improved solubility of recombinant proteins in *Escherichia coli*. *Gene* 440 9–15. 10.1016/j.gene.2009.03.011 19328840 PMC2683908

[B84] Lanz N. D. Blaszczyk A. J. McCarthy E. L. Wang B. Wang R. X. Jones B. S. (2018). Enhanced solubilization of class B radical S-adenosylmethionine methylases by improved cobalamin uptake in *Escherichia coli*. *Biochem* 57 1475–1490. 10.1021/acs.biochem.7b01205 29298049 PMC5941297

[B85] Lebendiker M. Danieli T. (2014). Production of prone-to-aggregate proteins. *FEBS Lett.* 588 236–246. 10.1016/j.febslet.2013.10.044 24211444

[B86] Lee D. H. Kim M. D. Lee W. H. Seo J. H. Kweon D. H. (2004). Consortium of fold-catalyzing proteins increases soluble expression of cyclohexanone monooxygenase in recombinant *Escherichia coli*. *Appl. Microbiol. Biotechnol.* 63 549–552. 10.1007/s00253-003-1370-z 12827321

[B87] Lewis M. (2013). Allostery and the lac operon. *J. Mol. Biol.* 425 2309–2316. 10.1016/j.jmb.2013.03.003 23500493

[B88] Li Y. Sun X. Bi Y. Ge Y. Wang Y. (2009). Antifungal activity of chitosan on *Fusarium sulphureum* in relation to dry rot of potato tuber. *Agric. Sci. China* 8 597–604. 10.1016/s1671-2927(08)60251-5

[B89] Lindner A. B. Madden R. Demarez A. Stewart E. J. Taddei F. (2008). Asymmetric segregation of protein aggregates is associated with cellular aging and rejuvenation. *PNAS* 105 3076–3081. 10.1073/pnas.0708931105 18287048 PMC2268587

[B90] Liovic M. Ozir M. Zavec A. B. Peternel S. Komel R. Zupancic T. (2012). Inclusion bodies as potential vehicles for recombinant protein delivery into epithelial cells. *Microb. Cell Fact.* 11:67. 10.1186/1475-2859-11-67 22624805 PMC3434093

[B91] Lobstein J. Emrich C. A. Jeans C. Faulkner M. Riggs P. Berkmen M. (2012). SHuffle, a novel *Escherichia coli* protein expression strain capable of correctly folding disulfide bonded proteins in its cytoplasm. *Microb. Cell Fact.* 11:56. 10.1186/1475-2859-11-56 PMC3526497 22569138

[B92] Loo Y. Goktas M. Tekinay A. B. Guler M. O. Hauser C. A. E. Mitraki A. (2015). Self-assembled proteins and peptides as scaffolds for tissue regeneration. *Adv. Healthc. Mater.* 4 2557–2586. 10.1002/adhm.201500402 26461979

[B93] Ma W. Chen K. Li Y. Hao N. Wang X. Ouyang P. (2017). Advances in cadaverine bacterial production and its applications. *Engineering* 3 308–317. 10.1016/j.eng.2017.03.012

[B94] Macholz R. (1989). 1-Butanol health and safety guide (a companion volume to environmental health criteria 65: butanols-four isomers: 1-butanol, 2-butanol. tert-butanol, isobutanol). *Food/Nahrung* 33 382–382. 10.1002/food.19890330432

[B95] Malik A. (2016). Protein fusion tags for efficient expression and purification of recombinant proteins in the periplasmic space of *E. coli*. *3 Biotech* 6:44. 10.1016/j.pep.2017.01.006 28330113 PMC4742420

[B96] Malys N. Wishart J. A. Oliver S. G. McCarthy J. E. G. (2011). Protein production in S. cerevisiae for systems biology studies. *Methods Enzymol.* 500 197–212. 10.1016/b978-0-12-385118-5.00011-6 21943899

[B97] Mamat U. Woodard R. W. Wilke K. Souvignier C. Mead D. Steinmetz E. (2013). Endotoxin-free protein production—ClearColiTM technology. *Nat. Methods* 10 916–916.

[B98] Manta B. Boyd D. Berkmen M. (2019). Disulfide bond formation in the periplasm of *Escherichia coli*. *EcoSal Plus* 8 1–20. 10.1128/ecosalplus.ESP-0012-2018 30761987 PMC11573287

[B99] Markossian K. A. Kurganov I. (2004). Protein folding, misfolding, and aggregation. Formation of inclusion bodies and aggresomes. *Biochemical* 69 971–984. 10.1023/b:biry.0000043539.07961.4c 15521811

[B100] Martelli G. P. Castellano M. A. (1971). Light and electron microscopy of the intracellular inclusions of cauliflower mosaic virus. *J. Gen. Virol.* 13 133–140. 10.1099/0022-1317-13-1-133 4108668

[B101] Mattheakis L. C. Bhattt R. R. Dower W. J. (1994). An in vitro polysome display system for identifying ligands from very large peptide libraries. *Proc. Natl. Acad. Sci. USA* 91 9022–9026. 10.1073/pnas.91.19.9022 7522328 PMC44739

[B102] Miroux B. Walker J. E. (1996). Over-production of proteins in *Escherichia coli*: mutant hosts that allow synthesis of some membrane proteins and globular proteins at high levels. *J. Mol. Biol.* 260 289–298. 10.1006/jmbi.1996.0399 8757792

[B103] Molinari M. (2007). N-glycan structure dictates extension of protein folding or onset of disposal. *Nat. Chem. Biol.* 3 313–320. 10.1038/nchembio880 17510649

[B104] Morell M. Bravo R. Espargaró A. Sisquella X. Avilés F. X. Fernàndez-Busquets X. (2008). Inclusion bodies: specificity in their aggregation process and amyloid-like structure. *Biochim. Biophys. ActaMol. Cell Res.* 1783 1815–1825. 10.1016/j.bbamcr.2008.06.007 18619498

[B105] Morimoto R. Kelly J. W. Hartl F. -U. (2019). *Protein Homeostasis*. Second Edition Cold Spring Harbour, N.Y: Cold Spring Harbor Laboratory Press.

[B106] Nahálka J. Vikartovská A. Hrabárová E. (2008). A crosslinked inclusion body process for sialic acid synthesis. *J. Biotechnol.* 134 146–153. 10.1016/j.jbiotec.2008.01.014 18313163

[B107] Navarro S. Ventura S. (2014). Fluorescent dye ProteoStat to detect and discriminate intracellular amyloid-like aggregates in *Escherichia coli*. *Biotechnol. J.* 9 1259–1266. 10.1002/biot.201400291 25112199

[B108] Ngiam C. Jeenes D. J. Punt P. J. Van Den Hondel C. A. M. J. J. Archer D. B. (2000). Characterization of a foldase, protein disulfide isomerase a, in the protein secretory pathway of *Aspergillus niger*. *Appl. Environ. Microbiol.* 66 775–782 10.1128/aem.66.2.775-782.2000 10653750 PMC91895

[B109] Nguyen V. D. Hatahet F. Salo K. E. H. Enlund E. Zhang C. Ruddock L. W. (2011). Pre-expression of a sulfhydryl oxidase significantly increases the yields of eukaryotic disulfide bond containing proteins expressed in the cytoplasm of *E.coli*. *Microb. Cell Fact.* 10:1. 10.1186/1475-2859-10-1 21211066 PMC3022669

[B110] Ni Y. Reye J. Chen R. R. (2007). Lpp deletion as a permeabilization method. *Biotechnol. Bioeng.* 97 1347–1356. 10.1002/bit.21375 17304571

[B111] Oganesyan N. Ankoudinova I. Kim S. -H. Kim R. (2007). Effect of osmotic stress and heat shock in recombinant protein overexpression and crystallization. *Protein Expr. Purif.* 52 280–285. 10.1016/j.pep.2006.09.015 17126029 PMC1865119

[B112] Palmer I. Wingfield P. T. (2004). Preparation and extraction of insoluble (inclusion-body) proteins from *Escherichia coli*. *Curr. Protoc. Protein Sci.* 1 1–25. 10.1002/0471140864.ps0603s38 PMC3518028 18429271

[B113] Papp E. Csermely P (2006). Chemical chaperones: mechanisms of action and potential use. *Handb. Exp. Pharmacol.* 172 405–416 10.1007/3-540-29717-0_16 16610368

[B114] Paraskevopoulou V. Falcone F. (2018). Polyionic tags as enhancers of protein solubility in recombinant protein expression. *Microorganisms* 6:47. 10.3390/microorganisms6020047 29882886 PMC6027335

[B115] Park J. H. Kwon H. W. Jeong K. J. (2013). Development of a plasmid display system with an Oct-1 DNA-binding domain suitable for in vitro screening of engineered proteins. *J. Biosci. Bioeng.* 116 246–252. 10.1016/j.jbiosc.2013.02.005 23490644

[B116] Park Y. Shin J. Yang J. Kim H. Jung Y. Oh H. (2020). Plasmid display for stabilization of enzymes inside the cell to improve whole-cell biotransformation efficiency. *Front. Bioeng. Biotechnol.* 7:444. 10.3389/fbioe.2019.00444 PMC6967079 31998709

[B117] Quan S. Koldewey P. Tapley T. Kirsch N. Ruane K. M. Pfizenmaier J. (2011). Genetic selection designed to stabilize proteins uncovers a chaperone called Spy. *Nat. Struct. Mol. Biol.* 18 262–269 10.1038/nsmb.2016 21317898 PMC3079333

[B118] Rajan R. S. Illing M. E. Bence N. F. Kopito R. R. (2001). Specificity in intracellular protein aggregation and inclusion body formation. *PNAS* 98 13060–13065. 10.1073/pnas.181479798 11687604 PMC60824

[B119] Ramón A. Señorale-Pose M. Marín M. (2014). Inclusion bodies: not that bad. *Front. Microbiol.* 5:56. 10.3389/fmicb.2014.00056 PMC3924032 24592259

[B120] Rasiah I. A. Rehm B. H. A. (2009). One-step production of immobilized α-amylase in recombinant *Escherichia coli*. *Appl. Environ. Microbiol.* 75 2012–2016. 10.1128/aem.02782-08 19201981 PMC2663195

[B121] Restrepo-Pineda S. Bando-Campos C. G. Valdez-Cruz N. A. Trujillo-Roldán M. A. (2019). Recombinant production of ESAT-6 antigen in thermoinducible *Escherichia coli*: the role of culture scale and temperature on metabolic response, expression of chaperones, and architecture of inclusion bodies. *Cell Stress Chaperones* 24 777–792. 10.1007/s12192-019-01006-x 31165436 PMC6629757

[B122] Rinas U. Bailey J. E. (1992). Protein compositional analysis of inclusion bodies produced in recombinant *Escherichia* coil. *Appl. Microbiol. Biotechnol.* 37 609–614. 1369400 10.1007/BF00240735

[B123] Rinas U. Garcia-Fruitós E. Corchero J. L. Vázquez E. Seras-Franzoso J. Villaverde A. (2017). Bacterial inclusion bodies: discovering their better half. *Trends Biochem. Sci.* 42 726–737. 10.1016/j.tibs.2017.01.005 28254353

[B124] Roberts R. W. Szostak J. W. (1997). RNA-peptide fusions for the in vitro selection of peptides and proteins. *PNAS* 94 12297–12302 10.1073/pnas.94.23.12297 9356443 PMC24913

[B125] Rosano G. L. Ceccarelli E. A. (2014). Recombinant protein expression in *Escherichia coli*: advances and challenges. *Front. Microbiol.* 5:172. 10.3389/fmicb.2014.00172 PMC4029002 24860555

[B126] Ruan A. Ren C. Quan S. (2020). Conversion of the molecular chaperone Spy into a novel fusion tag to enhance recombinant protein expression. *J. Biotechnol.* 307 131–138. 10.1016/j.jbiotec.2019.11.006 31705934

[B127] Rueda F. Cano-Garrida O. Mamat U. Wilke K. Sera-Franzoso J. García-Fruitós E. (2014). Production of functional inclusion bodies in endotoxin-free E.c*oli*. *Appl. Mircobiol. Biotechnol.* 98 9229–9238. 10.1007/s00253-014-6008-9 25129611

[B128] Sarch C. Suzuki H. Master E. R. Wang W. (2019). Kinetics and regioselectivity of three GH62 α-L-arabinofuranosidases from plant pathogenic fungi. *Biochim. Biophys. Acta Gen. Subj.* 1863 1070–1078. 10.1016/j.bbagen.2019.03.020 30936018

[B129] Schramm F. D. Schroeder K. Jonas K. (2019). Protein aggregation in bacteria. *FEMS Microbiol. Rev.* 44 54–72 10.1093/femsre/fuz026 PMC7053576 31633151

[B130] Selas Castiñeiras T. Williams S. G. Hitchcock A. Cole J. A. Smith D. C. Overton T. W. (2018). Development of a generic β-lactamase screening system for improved signal peptides for periplasmic targeting of recombinant proteins in *Escherichia coli*. *Sci. Rep.* 8:6986. 10.1038/s41598-018-25192-3 PMC5934370 29725125

[B131] Seras-Franzoso J. Dez-Gil C. Vazquez E. Garca-Fruits E. Cubarsi R. Ratera I. (2012). Bioadhesiveness and efficient mechanotransduction stimuli synergistically provided by bacterial inclusion bodies as scaffolds for tissue engineering. *Nanomedicine* 7 79–93. 10.2217/nnm.11.83 22142409

[B132] Shao F. Bader M. W. Jakob U. Bardwell J. C. A. (2000). DsbG, a protein disulfide isomerase with chaperone activity. *J. Biol. Chem.* 275 13349–13352. 10.1074/jbc.275.18.13349 10788443

[B133] Shen D. Coleman J. Chan E. Nicholson T. P. Dai L. Sheppard P. W. (2011). Novel cell- and tissue-based assays for detecting misfolded and aggregated protein accumulation within aggresomes and inclusion bodies. *Cell Biochem. Biophys.* 60 173–185. 10.1007/s12013-010-9138-4 21132543 PMC3112480

[B134] Shin H. D. Chen R. R. (2008). Extracellular recombinant protein production from an *Escherichia coli* lpp deletion mutant. *Biotechnol. Bioeng.* 101 1288–1296. 10.1002/bit.22013 18781683

[B135] Shirano Y. Shibata D. (1990). Low temperature cultivation of *Escherichia coli* carrying a rice lipoxygenase L-2 cDNA produces a soluble and active enzyme at a high level. *FEBS Lett.* 271 128–130. 10.1016/0014-5793(90)80388-y 2121525

[B136] Sina M. Farajzadeh D. Dastmalchi S. (2015). Effects of environmental factors on soluble expression of a humanized anti-TNF-α scFv antibody in *Escherichia coli*. *Adv. Pharm. Bull.* 5 455–461. 10.15171/apb.2015.062 26819916 PMC4729350

[B137] Singh A. Upadhyay V. Singh A. Panda A. K. (2020). Structure-function relationship of inclusion bodies of a multimeric protein. *Front. Microbiol.* 11:876. 10.3389/fmicb.2020.00876 PMC7225587 32457730

[B138] Singh A. Upadhyay V. Upadhyay A. K. Singh S. M. Panda A. K. (2015). Protein recovery from inclusion bodies of *Escherichia coli* using mild solubilization process. *Microb. Cell Fact.* 14:41. 10.1186/s12934-015-0222-8 PMC4379949 25889252

[B139] Singhvi P. Saneja A. Srichandan S. Panda A. K. (2020). Bacterial inclusion bodies: a treasure trove of bioactive proteins. *Trends Biotechnol.* 38 474–486. 10.1016/j.tibtech.2019.12.011 31954528

[B140] Slouka C. Kopp J. Spadiut O. Herwig C. (2019). Perspectives of inclusion bodies for bio-based products: curse or blessing? *Appl. Microbiol. Biotechnol.* 103 1143–1153. 10.1007/s00253-018-9569-1 30569219 PMC6394472

[B141] Smith G. P. (1985). Filamentous fusion phage: novel expression vectors that display cloned antigens on the virion surface. *Science* 228 1315–1317. 10.1126/science.4001944 4001944

[B142] Sohail A. A. Gaikwad M. Khadka P. Saaranen M. J. Ruddock L. W. (2020). Production of extracellular matrix proteins in the cytoplasm of *E. coli*: making giants in tiny factories. *Int. J. Mol. Sci.* 21:688. 10.3390/ijms21030688 31973001 PMC7037224

[B143] Stargardt P. Feuchtenhofer L. Cserjan-Puschmann M. Striedner G. Mairhofer J. (2020). Bacteriophage inspired growth-decoupled recombinant protein production in *Escherichia coli*. *ACS Synth. Biol.* 9 1336–1348. 10.1021/acssynbio.0c00028 32324989

[B144] Stirling P. C. Lundin V. F. Leroux M. R. (2003). Getting a grip on non-native proteins. *EMBO Rep.* 4 565–570. 10.1038/sj.embor.embor869 12776175 PMC1319208

[B145] Strandberg L. Enfors S. -O. (1991). Factors influencing inclusion body formation in the production of a fused protein in *Escherichia coli*. *Appl. Environ. Microbiol.* 57 1669–1674. 10.1128/aem.57.6.1669-1674.1991 1908208 PMC183450

[B146] Stull F. Betton J. -M. Bardwell J. C. A. (2018). Periplasmic chaperones and prolyl isomerases. *EcoSal Plus* 8 1–16. 10.1128/ecosalplus.ESP-0005-2018 29988001 PMC11575675

[B147] Thomas J. G. Baneyx F. (1996). Protein folding in the cytoplasm of *Escherichia coli*: requirements for the DnaK-DnaJ-GrpE and GroEL-GroES molecular chaperone machines. *Mol. Microbiol.* 21 1185–1196 10.1046/j.1365-2958.1996.651436.x 8898387

[B148] Tolia N. H. Joshua-Tor L. (2006). Strategies for protein coexpression in *Escherichia coli*. *Nat. Methods* 3 55–64. 10.1038/nmeth0106-55 16369554

[B149] Tong Y. Feng S. Xin Y. Yang H. Zhang L. Wang W. (2016). Enhancement of soluble expression of codon-optimized Thermomicrobium roseum sarcosine oxidase in *Escherichia coli* via chaperone co-expression. *J. Biotechnol.* 218 75–84. 10.1016/j.jbiotec.2015.11.018 26626227

[B150] Tsolis A. C. Papandreou N. C. Iconomidou V. A. Hamodrakas S. J. (2013). A consensus method for the prediction of “aggregation-prone” peptides in globular proteins. *PLoS One* 8:e54175. 10.1371/journal.pone.0054175 23326595 PMC3542318

[B151] Tsumoto K. Ejima D. Kumagai I. Arakawa T. (2003). Practical considerations in refolding proteins from inclusion bodies. *Protein Expr. Purif.* 28 1–8. 10.1016/s1046-5928(02)00641-1 12651100

[B152] Upadhyay A. K. Murmu A. Singh A. Panda A. K. (2012). Kinetics of inclusion body formation and its correlation with the characteristics of protein aggregates in *Escherichia coli*. *PLoS One* 7: e33951. 10.1371/journal.pone.0033951 22479486 PMC3315509

[B153] Valax P. Georgiou G. (1993). Molecular characterization of β-lactamase inclusion bodies produced in *Escherichia coli*. 1. *Composition. Biotechnol. Prog.* 9 539–547. 10.1021/bp00023a014 7764166

[B154] Vázquez E. Corchero J. L. Burgueño J. F. Seras-Franzoso J. Kosoy A. Bosser R. (2012). Functional inclusion bodies produced in bacteria as naturally occurring nanopills for advanced cell therapies. *Adv. Mater.* 24 1742–1747. 10.1002/adma.201104330 22410789

[B155] Villaverde A. Corchero J. L. Seras-Franzoso J. Garcia-Fruitós E. (2015). Functional protein aggregates: just the tip of the iceberg. *Nanomedicine* 10 2881–2891. 10.2217/nnm.15.125 26370294

[B156] Vu T. T. Jeong B. Yu J. Koo B. K. Jo S. H. Robinson R. C. (2014). Soluble prokaryotic expression and purification of crotamine using an N-terminal maltose-binding protein tag. *Toxicon* 92 157–165. 10.1016/j.toxicon.2014.10.017 25448388

[B157] Wacker M. Linton D. Hitchen P. G. Nita-Lazar Y. Haslam M. North S. M. (2002). N-linked glycosylation in *Campylobacter jejuni* and its functional transfer into *E. coli*. *Science* 298 1790–1793. 10.1126/science.298.5599.1790 12459590

[B158] Wagner S. Klepsch M. M. Schlegel S. Appel A. Draheim R. Tarry M. (2008). Tuning *Escherichia coli* for membrane protein overexpression. *Proc. Natl. Acad. Sci. U. S. A.* 105 14371–14376. 18796603 10.1073/pnas.0804090105 PMC2567230

[B159] Walsh G. (2010). Post-translational modifications of protein biopharmaceuticals. *Drug Discov. Today* 15 773–780. 10.1016/j.drudis.2010.06.009 20599624

[B160] Walsh I. Seno F. Tosatto S. C. E. Trovato A. (2014). PASTA 2.0: an improved server for protein aggregation prediction. *Nucleic Acids Res.* 42 W301–W307. 24848016 10.1093/nar/gku399 PMC4086119

[B161] Wang L. (2009). Towards revealing the structure of bacterial inclusion bodies. *Prion* 3 139–145. 10.4161/pri.3.3.9922 19806034 PMC2802778

[B162] Wang W. Andric N. Sarch C. Silva B. T. Tenkanen M. Master E. R. (2018). Constructing arabinofuranosidases for dual arabinoxylan debranching activity. *Biotechnol. Bioeng.* 115 41–49. 10.1002/bit.26445 28868788

[B163] Wang W. Archbold T. Lam J. S. Kimber M. S. Fan M. Z. (2019). A processive endoglucanase with multi-substrate specificity is characterized from porcine gut microbiota. *Sci. Rep.* 9:13630. 10.1038/s41598-019-50050-1 PMC6754456 31541154

[B164] Wang W. Yan R. Nocek B. P. Vuong T. V. Leo R. Di, Xu X. (2016). Biochemical and structural characterization of a five-domain GH115 α-Glucuronidase from the marine bacterium Saccharophagus degradans 2-40T. *J. Biol. Chem.* 291 14120–14133. 10.1074/jbc.m115.702944 27129264 PMC4933171

[B165] Winkler J. Seybert A. König L. Pruggnaller S. Haselmann U. Sourjik V. (2010). Quantitative and spatio-temporal features of protein aggregation in *Escherichia coli* and consequences on protein quality control and cellular ageing. *EMBO J.* 29 910–923. 10.1038/emboj.2009.412 20094032 PMC2837176

[B166] Xiong S. Wang Y.-F. Ren X.-R. Li B. Zhang M.-Y. Luo Y. (2005). Solubility of disulfide-bonded proteins in the cytoplasm of *Escherichia coli* and its “oxidizing” mutant. *World J. Gastroenterol.* 11 1077–1082 10.3748/wjg.v11.i7.1077 15742420 PMC4250777

[B167] Yakupova E. I. Bobyleva L. G. Vikhlyantsev I. M. Bobylev A. G. (2019). Congo Red and amyloids: history and relationship. *Biosci. Rep.* 39:BSR20181415. 10.1042/BSR20181415 PMC6331669 30567726

[B168] Yang X. Zhang Y. (2013). Effect of temperature and sorbitol in improving the solubility of carboxylesterases protein CpCE-1 from *Cydia pomonella* and biochemical characterization. *Appl. Microbiol. Biotechnol.* 97 10423–10433. 10.1007/s00253-013-5236-8 24072161

[B169] Yoshimura Y. Lin Y. Yagi H. Lee Y. -H. Kitayama H. Sakurai K. (2012). Distinguishing crystal-like amyloid fibrils and glass-like amorphous aggregates from their kinetics of formation. *Proc. Natl. Acad. Sci. U. S. A.* 109 14446–14451. 10.1073/pnas.1208228109 22908252 PMC3437889

[B170] Yumerefendi H. Tarendeau F. Mas P. J. Hart D. J. (2010). ESPRIT: an automated, library-based method for mapping and soluble expression of protein domains from challenging targets. *J. Struct. Biol.* 172 66–74. 10.1016/j.jsb.2010.02.021 20206698

[B171] Zeng H. Yang A. (2019). Quantification of proteomic and metabolic burdens predicts growth retardation and overflow metabolism in recombinant *Escherichia coli*. *Biotechnol. Bioeng.* 116 1484–1495. 10.1002/bit.26943 30712260

[B172] Žerovnik E. Škarabot M. Škerget K. Giannini S. Stoka V. Jenko-Kokalj S. (2007). Amyloid fibril formation by human stefin B: influence of pH and TFE on fibril growth and morphology. *Amyloid* 14 237–247. 10.1080/13506120701461137 17701471

[B173] Zhang W. Lu J. Zhang S. Liu L. Pang X. Lv J. (2018). Development an effective system to expression recombinant protein in *E. coli* via comparison and optimization of signal peptides: expression of *Pseudomonas fluorescens* BJ-10 thermostable lipase as case study. *Microb. Cell Fact.* 17:50. 10.1186/s12934-018-0894-y PMC5872382 29592803

[B174] Zhuo X. F. Zhang Y. Y. Guan Y. X. Yao S. J. (2014). Co-expression of disulfide oxidoreductases DsbA/DsbC markedly enhanced soluble and functional expression of reteplase in *Escherichia coli*. *J. Biotechnol.* 192 197–203. 10.1016/j.jbiotec.2014.10.028 25449110

